# Adaptation of Arginine Synthesis among Uropathogenic Branches of the Escherichia coli Phylogeny Reveals Adjustment to the Urinary Tract Habitat

**DOI:** 10.1128/mBio.02318-20

**Published:** 2020-09-29

**Authors:** Michael E. Hibbing, Karen W. Dodson, Vasilios Kalas, Swaine L. Chen, Scott J. Hultgren

**Affiliations:** a Department of Molecular Microbiology and Center for Women’s Infectious Disease Research, Washington University School of Medicine, St. Louis, Missouri, USA; b Department of Medicine, Yong Loo Lin School of Medicine, National University of Singapore and Genome Institute of Singapore, Singapore; The Ohio State University School of Medicine

**Keywords:** *Escherichia coli*, arginine metabolism, positive selection, urinary tract infection

## Abstract

Urinary tract infections (UTIs) are predominantly caused by uropathogenic Escherichia coli (UPEC). UPEC pathogenesis requires passage through a severe population bottleneck involving intracellular bacterial communities (IBCs) that are clonal expansions of a single invading UPEC bacterium in a urothelial superficial facet cell. IBCs occur only during acute pathogenesis. The bacteria in IBCs form the founder population that develops into persistent extracellular infections. Only a small fraction of UPEC organisms proceed through the IBC cycle, regardless of the inoculum size. This dramatic reduction in population size precludes the utility of genomic mutagenesis technologies for identifying genes important for persistence. To circumvent this bottleneck, we previously identified 29 positively selected genes (PSGs) within UPEC and hypothesized that they contribute to virulence. Here, we show that 8 of these 29 PSGs are required for fitness during persistent bacteriuria. Conversely, 7/8 of these PSG mutants showed essentially no phenotype in acute UTI. Deletion of the PSG *argI* leads to arginine auxotrophy. Relative to the other *arg* genes, *argI* in the B2 clade (which comprises most UPEC strains) of E. coli has diverged from *argI* in other E. coli clades. Replacement of *argI* in a UPEC strain with a non-UPEC *argI* allele complemented the arginine auxotrophy but not the persistent bacteriuria defect, showing that the UPEC *argI* allele contributes to persistent infection. These results highlight the complex roles of metabolic pathways during infection and demonstrate that evolutionary approaches can identify infection-specific gene functions downstream of population bottlenecks, shedding light on virulence and the genetic evolution of pathogenesis.

## Abstract (precis)

Uropathogenic Escherichia coli (UPEC) is the most common cause of human urinary tract infection (UTI). Population bottlenecks during early stages of UTI make high-throughput screens impractical for understanding clinically important later stages of UTI, such as persistence and recurrence. As UPEC is hypothesized to be adapted to these later pathogenic stages, we previously identified 29 genes evolving under positive selection in UPEC. Here, we found that 8 of these genes, including *argI* (which is involved in arginine biosynthesis), are important for persistence in a mouse model of UTI. Deletion of *argI* and other arginine synthesis genes resulted in (i) arginine auxotrophy and (ii) defects in persistent UTI. Replacement of a B2 clade *argI* with a non-B2 clade *argI* complemented arginine auxotrophy, but the resulting strain remained attenuated in its ability to cause persistent bacteriuria. Thus, *argI* may have a second function during UTI that is not related to simple arginine synthesis. This study demonstrates how variation in metabolic genes can impact virulence and provides insight into the mechanisms and evolution of bacterial virulence.

## INTRODUCTION

Urinary tract infections (UTIs) are among the most common bacterial infections of humans, affecting over half of all women and accounting for ∼$2.5 billion in annual health care-related expenses in the United States (1, 2). Uropathogenic Escherichia coli (UPEC) accounts for the majority of community-acquired UTIs, typically acute uncomplicated cystitis (2, 3). Unfortunately, after an initial acute UTI, ∼25% of women experience at least one episode of recurrent UTI (rUTI) within 6 months of the initial infection (4, 5). While in some patients acute UTIs resolve within a week, other patients can develop chronic recurrent cystitis even despite treatment (6). Further, frequent empirical antimicrobial treatment of acute UTIs and prophylactic antimicrobial treatment to prevent rUTIs has resulted in the emergence and global dissemination of multidrug-resistant UPEC (7–9).

Experimental mouse models of UTI are able to mimic many of the known clinical manifestations of human UTIs, including acute cystitis, recurrent UTI, and chronic cystitis (10–14). During acute UTI, UPEC employs the FimH adhesin of the type 1 pilus to invade urothelial superficial facet cells via an endocytic mechanism (10–15). Following invasion, some UPEC organisms evade host cell exocytic mechanisms, escape the endocytic vesicle, and rapidly replicate to form dense clusters (∼1,000 to 10,000 CFU/epithelial cell) of clonal cytoplasmic bacteria, termed intracellular bacterial communities (IBCs) (16–21). IBCs are resistant to antibiotic treatment and the host immune response. In addition, quiescent intracellular reservoirs (QIRs) can form in the underlying transitional epithelial cells, comprising clusters of 2 to 12 membrane-bound bacteria that are dormant and thereby tolerant of antibiotics and host defenses (22–24). QIRs can persist for weeks to months, during which time the urine may be sterile; however, bacteria can reemerge from a QIR to cause a rUTI (25–27). Importantly, structures similar to IBCs can be found in the urine of human patients suffering from UTI, further supporting the relevance of experiments with the mouse model (28–31).

Mice with a history of UTI are predisposed to chronic rUTI. In mice, chronic cystitis occurs downstream of the IBC bottleneck and is defined as an extracellular infection characterized by persistent, high-titer bacteriuria (>10^4^ CFU/ml) and bladder colonization (>10^4^ CFU/bladder), chronic pyuria, ablation of the terminally differentiated superficial cells, urothelial hyperplasia, lymphoid follicles, and urothelial necrosis persisting for weeks (10). Mice with a history of chronic cystitis can be cured by antibiotic treatment; however, they are thereafter sensitized to higher rates of rUTI in subsequent challenge infections due to bladder remodeling (10, 13, 14). In mice, infection can leave a molecular imprint resulting in hundreds of differentially expressed genes compared to the bladders of naive mice; this physiological remodeling results in a predisposition to rUTI (13). Of note, in humans, the history of UTI is one of the most significant risk factors predisposing to a rUTI (32, 33).

One of the main experimental hurdles in studying factors important in persistent UTI and rUTI is the progressive population bottleneck that begins during the initial acute phase of cystitis. The bacteria within an IBC are clonal (each IBC is descended from a single invasive UPEC organism), and only 1 of 10^5^ to 10^6^ experimentally inoculated UPEC organisms forms an IBC (16). Therefore, IBC formation represents one severe population bottleneck that occurs during acute infection (16). The formation of QIRs and development of chronic cystitis incur additional bottlenecks, as measured by random loss of coinoculated, isogenic, differentially marked strains (16). The mechanisms causing these bottlenecks are not fully defined, but they are likely related to some combination of bacterial invasion, immune cell killing, intracellular innate immune responses, competitive growth, and continuous micturition. Because modern unbiased genetic approaches (like signature-tagged mutagenesis or transposon insertion sequencing [Tn-seq] screens) require testing of high-complexity mutant libraries (i.e., those having a large number of genetically distinct clones), population bottlenecks (which cause random loss of clones) prevent identification of mutants that exhibit defects downstream of the bottleneck (34–36). Recently, unbiased global approaches to interrogate UPEC, including comparative genomics, transcriptomics, and proteomics, have also been applied to analyze factors required for UTI pathogenesis (37–41), though these have not addressed chronic rUTI.

*In silico* sequence-based methods provide an alternative approach to identifying virulence factors that does not suffer from experimental population bottlenecks. UPEC are hypothesized to be better adapted to the urinary tract than non-UPEC strains. Any adaptive mutation(s) should be evolving under positive selection; therefore, we and others have conceptually connected loci under positive selection with virulence-related genes (42–49). Indeed, FimH, a critical virulence factor for acute UTI and IBC formation, is evolving under positive selection in UPEC, and identification of individually selected amino acids has led to fundamental insights into the structure, function, and evolution of FimH and the importance of a finely tuned conformational equilibrium to its function in UTIs (48–50). Thus, positive-selection analysis can identify virulence factors (and their functions) that otherwise might be difficult to study due to population bottlenecks.

We previously used an *in silico* comparative genomics analysis to identify 29 genes evolving under positive selection (here referred to as positively selected genes [PSGs]) specifically in UPEC but not other types of E. coli (47). These 29 PSGs are predicted to contribute to a diverse set of cellular functions, including iron uptake, membrane protein localization, DNA structure and repair, small-molecule import/export, cell division, and arginine metabolism (47); however, their roles in chronic UTI have not been characterized. Here, we tested the impact of these 29 PSGs on the fitness of UPEC in a mouse model of UTI, focusing on chronic cystitis (10–12, 51). In total, single gene deletions of 10 different PSGs had lower fitness in at least one of our *in vivo* infection models, with 8 single PSG deletions showing defects during chronic cystitis. Further analysis of the *argI* gene (encoding the anabolic ornithine transcarbamoylase) showed that while arginine biosynthesis is important for *in vivo* fitness, *argI* also seems to have an additional *in vivo* function during UTI. Specifically, an allelic replacement of a clade B2 *argI* with a non-B2 *argI* allele complements arginine auxotrophy but not *in vivo* virulence. In keeping with this, *argI* from the B2 clade, which comprises the majority of UPEC strains, is highly diverged from the *argI* genes found in other E. coli clades (39, 52, 53). Together, these results reveal an unexpected complexity in the importance of arginine during chronic UTI, which could not have been found from traditional genomic mutagenesis studies due to the population bottlenecks occurring during *in vivo* UTI. They also highlight the value of positive-selection analysis, which has now identified *in vivo*-specific roles mediated by allelic variation in both *fimH* and *argI*. Finally, our results suggest that screening for anomalous divergence patterns, a rapid technique amenable to modern genomic data sets, may be a useful approach to identify other genes involved in virulence and bacterial evolution.

## RESULTS

### PSG mutations have little effect on *in vitro* phenotypes.

**FIG 1:**
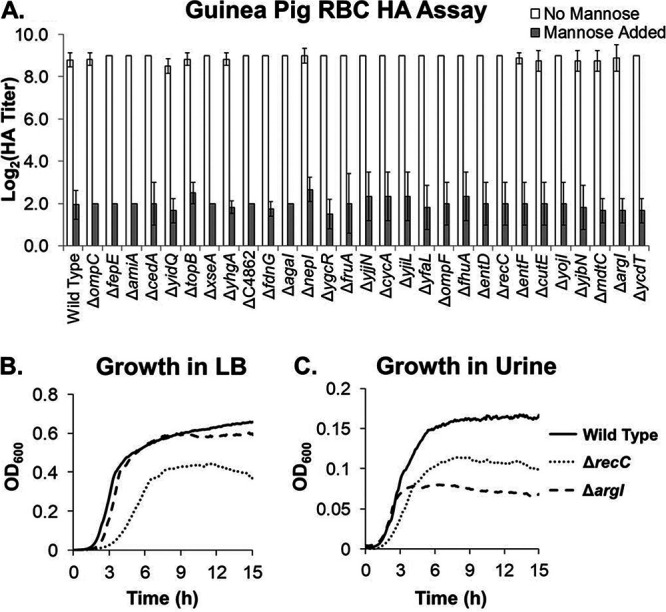
PSG mutants with growth defects. (A) Mannose-sensitive (phosphate-buffered saline [PBS] only) and mannose-insensitive (PBS + 4% methyl α-d-mannopyranoside) HA titers of the PSG mutants. HA titers are the means and standard deviations for ≥3 biological replicates. Columns marked with an asterisk are significantly different from the wild-type value by Student's *t* test with a cutoff *P* value of >0.05. (B) The Δ*recC* mutant has growth rate and yield defects when grown in LB medium at 37°C with aeration. (C) Both the Δ*recC* and the Δ*argI* mutant have growth defects when grown in filter-sterilized human urine pooled from multiple donors at 37°C with aeration. The Δ*recC* mutant has growth rate and yield defects, while the Δ*argI* mutant has a growth yield defect. Growth curves are the means of 4 technical replicates and are representative of experiments conducted on different days and with different batches of LB or urine.

The primary hypothesis is that genes under positive selection in UPEC play a role during UTI *in vivo*. We created knockouts in each of the 29 previously identified PSGs in UTI89, a prototypical UPEC clinical isolate. We first tested these mutants for *in vitro* phenotypes previously shown to be associated with pathogenesis: (i) type 1 pilus expression and function as measured by guinea pig red blood cell hemagglutination (HA) assay and (ii) growth in rich (LB) medium and in pooled filtered human urine (54–56). All the PSG mutants were indistinguishable from the wild type in all assays with two exceptions (Fig. 1A to C): (i) the Δ*recC* mutant had a general growth defect in both LB and urine (Fig. 1B and C), and (ii) the Δ*argI* mutant had a mild growth defect in urine (but not in LB), wherein the cell density during stationary phase was lower (Fig. 1B and C). Supporting these observations, deletion of either *recC* or *argI* in the UPEC isolate CFT073 was recently shown to cause an *in vitro* growth defect in human urine (36).

### Multiple PSGs have a role in chronic UTI.

**FIG 2:**
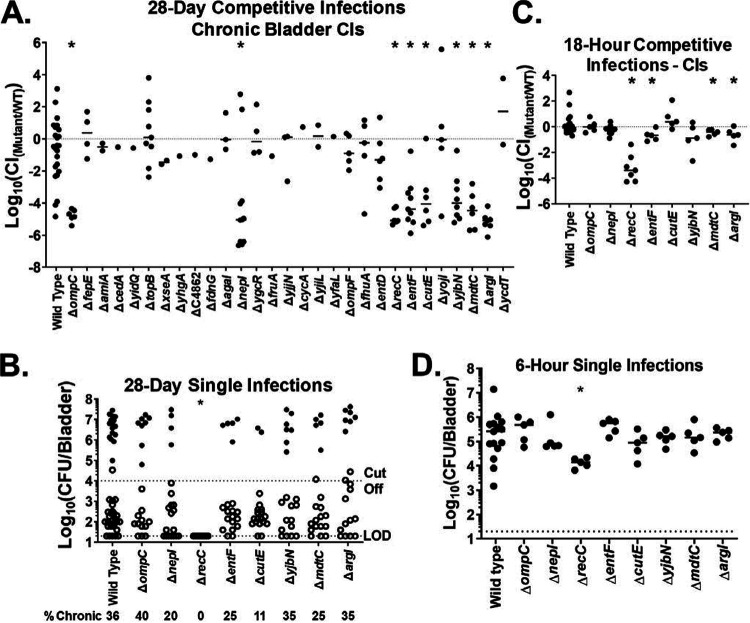
*In vivo* effects of the PSG mutations. (A) Competitive outcomes of wild-type versus PSG mutant chronic coinfections at 28 dpi. Circles represent the bladder of a single mouse with chronic cystitis, defined as persistent high-titer bacteriuria (>10^4^ CFU/ml). The horizontal line indicates the median log_10_(CI). An asterisk indicates that the median log_10_(CI) is significantly different from the median log_10_(CI) of the control (wild type versus wild type) by the Mann-Whitney U test (*P* < 0.05). Mice that had 1 or more urine samples with low-titer bacteriuria (<10^4^ CFU/ml) were analyzed separately, and those data are displayed along with the raw CFU totals and all urine and kidney data in the supplemental material (Fig. S1 and S2). The total number of infected mice and the frequency of chronic cystitis of each competitive infection is displayed in the supplemental material (Table S1). In the initial screen (A), tests with mutants that did not clearly exhibit competitive defects in a 5-mouse group were not repeated due to ethical concerns. Mutants that did exhibit competitive defects in the initial screen were repeat tested with at least 1 biological replicate of 10 mice. (B) Bladder colonization and frequency of chronicity caused by the PSG mutants in single infections at 28 dpi. Closed circles represent a single mouse with persistent high-titer bacteriuria (>10^4^ CFU/ml). Open circles represent a mouse with at least 1 urine sample with low-titer bacteriuria (<10^4^ CFU/ml). The frequency of chronicity for each PSG mutant was determined by dividing the number of mice with persistent high-titer bacteriuria by the total number of mice infected. An asterisk indicates that the frequency of chronicity is significantly different from that of the wild type by Fisher’s exact test (*P* < 0.05). At least two biological replicates with 10 mice each were tested for each strain except the Δ*recC* mutant, which was tested with only one biological replicate of 10 mice. Raw CFU and all urine and kidney data are displayed in the supplemental material (Fig. S3). (C) Competitive outcomes of wild-type versus PSG mutant coinfections at 18 hpi. Circles represent a single mouse. The horizontal line indicates the median log_10_(CI). An asterisk indicates that the median log_10_(CI) is significantly different from the median log_10_(CI) of the control (wild type versus wild type) by the Mann-Whitney U test (*P* < 0.05). A single biological replicate of 5 to 7 mice was used for each competition except for the wild-type–versus–wild-type comparison, which was carried out a second time. Raw CFU totals and all urine and kidney data are displayed in the supplemental material (Fig. S4). (D) Bladder colonization by the PSG mutants in single infections at 6 hpi. Circles represent a single mouse. The horizontal line indicates the median CFU/bladder. An asterisk indicates that the median CFU/bladder is significantly different from that of the wild type by the Mann-Whitney U test (*P* < 0.05). A single biological replicate of 5 mice was conducted for each strain except for the experiment with the wild type, which was carried out a total of three times. Kidney CFU data are displayed in the supplemental material (Fig. S5).

We next tested the competitive fitness of each PSG mutant in a mouse model of chronic UTI, in which naive female 7- to 8-week-old C3H/HeN mice were inoculated with a mixture of ∼10^8^ CFU each of a PSG mutant and wild-type UTI89. Infected mice were monitored by urinalysis until 28 days post-infection (dpi), at which time the mice were sacrificed and bladder and kidney burdens were determined (Fig. 2A and Fig. S1 and S2). It was previously shown that these infections result in two different UTI outcomes: (i) high-titer and chronic infection and (ii) resolution with or without the formation of QIRs (10). These outcomes are reliably distinguished by persistently high urine titers (>10^4^ CFU/ml), indicating chronic infection, and low or variable urine titers, indicating resolution; we therefore analyzed the data for mice with chronic and resolved infections separately (Fig. 2A and Fig. S1 and S2). Eight PSG mutants had chronic cystitis defects (*ompC*, *nepI* [previously *yicM* {57}], *recC*, *entF*, *cutE*, *yjbN*, *mdtC* [previously *yegO* {58}], and *argI*), with all being dramatically outcompeted by the wild type, with median log_10_(CI) (competitive index) values below −4 at 28 dpi (indicating that there are >10,000 times more wild-type than mutant bacteria) (Fig. 2A). Six PSG mutants had fitness defects in the resolved group (*amiA*, *entD*, *cutE*, *yjbN*, *mdtC*, and *argI*), though due to the lower overall titers, the median log_10_(CI) values ranged between −0.2 and −1.29 at 28 dpi (Fig. S1 and S2). Notably, as strains with mutations in 4 PSGs (*cutE*, *yjbN*, *mdtC*, and *argI*) had defects in mice with chronic and resolved infections, there were a total of 10 PSGs that contributed to some form of long-term persistence in the urinary tract (Fig. 2A and Fig. S2). Interestingly, single-infection assays showed that, when inoculated individually into mice, all PSG mutants tested achieved bacterial titers indistinguishable from that of the wild type at 28 dpi, again with the exception of the Δ*recC* mutant (Fig. 2B).

10.1128/mBio.02318-20.1 FIG S1 Full PSG mutant CFU data and infection time course. Infection and competitive outcomes of 28-day infections with equal numbers of differentially marked wild-type and PSG mutant UTI89. CFU graphs show the total bacteria recovered from the urine and tissues of the infections at the indicated time points. The cutoff indicates the level of persistent bacteriuria necessary for an infection to be classified as chronic cystitis. Chronic-infection CI graphs show the log_10_(CI_(mutant/wild type)_) from mice with persistent high-titer bacteriuria (>10^4^ CFU/ml urine). Resolved-infection CI graphs show the log_10_(CI_(mutant/wild type)_) from mice with at least one incidence of low-titer bacteriuria (<10^4^ CFU/ml urine). Download FIG S1, PDF file, 2.9 MB. Copyright © 2020 Hibbing et al. 2020 Hibbing et al. This content is distributed under the terms of the Creative Commons Attribution 4.0 International license.

10.1128/mBio.02318-20.2 FIG S2 *In vivo* effects of the PSG mutations in mice that resolved their infection. Competitive outcomes of wild-type–versus–PSG-mutant chronic coinfections at 28 dpi. Circles represent the log_10_(CI_(mutant/wild type)_) from the bladder of a single mouse with a resolved UTI, defined as at least one sample with low-titer bacteriuria (<10^4^ CFU/ml). Horizontal lines indicate the median log_10_(CI_(mutant/wild type)_). An asterisk indicates that the median log_10_(CI_(mutant/wild type)_) is significantly different from the median log_10_(CI_(mutant/wild type)_) of the control (wild type versus wild type) by the Mann-Whitney U test (*P* < 0.05). Download FIG S2, PDF file, 0.1 MB. Copyright © 2020 Hibbing et al. 2020 Hibbing et al. This content is distributed under the terms of the Creative Commons Attribution 4.0 International license.

10.1128/mBio.02318-20.3 FIG S3 Outcomes of 28-day single infections with the PSG mutants. The total bacteria recovered from the urine and tissues of the infections at the indicated time points. The cutoff indicates the level of persistent bacteriuria necessary for an infection to be classified as chronic cystitis. Download FIG S3, PDF file, 0.4 MB. Copyright © 2020 Hibbing et al. 2020 Hibbing et al. This content is distributed under the terms of the Creative Commons Attribution 4.0 International license.

10.1128/mBio.02318-20.4 FIG S4 Urine, bladder, and kidney data of 18-h competitive infections. Infection and competitive outcomes from the urine, bladders, and kidneys of mice infected with equal numbers of differentially marked wild-type and PSG mutant bacteria at 18 hpi. CFU graphs show the total bacteria recovered from the urine and tissues of the infected mice. CI graphs show the log_10_(CI_(mutant/wild type)_) in the urine and tissues of the infected mice. An asterisk indicates values that are significantly different from the control (wild type versus wild type) by the Mann-Whitney U test (*P* < 0.05). Horizontal bars indicate the median CFU or CI values as appropriate. Download FIG S4, PDF file, 0.2 MB. Copyright © 2020 Hibbing et al. 2020 Hibbing et al. This content is distributed under the terms of the Creative Commons Attribution 4.0 International license.

10.1128/mBio.02318-20.5 FIG S5 Kidney colonization by the PSG mutants in single infections at 6 hpi. Kidney bacterial burdens at 6 hpi. Horizontal bars indicate median CFU/kidney pair. An asterisk indicates that the median CFU/kidney pair is significantly different from the wild type by the Mann-Whitney U test (*P* < 0.05). Download FIG S5, PDF file, 0.1 MB. Copyright © 2020 Hibbing et al. 2020 Hibbing et al. This content is distributed under the terms of the Creative Commons Attribution 4.0 International license.

10.1128/mBio.02318-20.10 TABLE S1 Total numbers of mice and frequency of outcome of 28-day competitive infections. Download Table S1, DOCX file, 0.01 MB. Copyright © 2020 Hibbing et al. 2020 Hibbing et al. This content is distributed under the terms of the Creative Commons Attribution 4.0 International license.

### PSG mutants affecting chronic infection have little effect on acute UTI.

The longitudinal urinalysis conducted during the competitive chronic infections revealed that the median log_10_(CI)s of the 8 PSG mutants with chronic cystitis defects were generally close to 0 for the first 24 h (i.e., indistinguishable from that of the wild type) (Fig. S1). We therefore tested the fitness of the 8 mutant strains in acute-cystitis experiments: single infections at 6 hpi and competitive infections at 18 hpi (Fig. 2C and D). Six of the eight mutants were indistinguishable from the wild type in these acute infections. The most pronounced exception was the Δ*recC* mutant, which showed a substantial defect in both assays (and which was also the mutant with the most severe *in vitro* growth defects). Furthermore, consistent with having a mild growth defect *in vitro* in urine, the *argI* mutant had a statistically significant, but quantitatively small, defect in these acute-infection assays. Interestingly, it was recently found that, in CFT073, deletion of either *recC* or *yojI* resulted in an acute fitness defect (36).

### *De novo* arginine synthesis is required during competitive chronic UTI.

**FIG 3:**
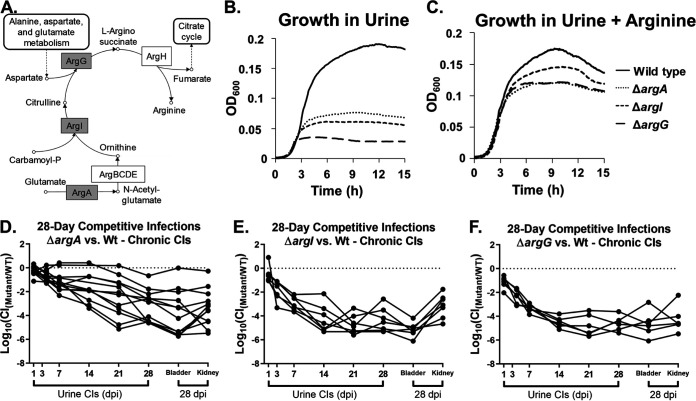
Arginine auxotrophs have competitive defects during chronic cystitis. (A) Simplified schematic of the conversion of glutamate to arginine (47, 64). Gray boxes indicate the enzymes coded for by the genes that were deleted in this analysis. (B and C) Growth of arginine synthesis mutants in pooled, filtered human urine (B) and pooled, filtered human urine supplemented 10 mM arginine (C). (D to F) Competitive colonization in the urinary tract of mice coinfected with wild-type UTI89 and either Δ*argA*, (D), Δ*argI* (E), or Δ*argG* (F) mutants as indicated by the log_10_(CI) in the urine during the 28-day infection and in the bladder and kidney tissue at sacrifice 28 dpi. Only mice with persistent bacteriuria of >10^4^ CFU/ml were included in the analysis. Median bladder CIs of all three mutants are significantly different from a hypothetical value of 0.0 by the Wilcoxon signed-rank test (*P* < 0.05). Raw CFU and the log_10_(CI) values of the resolved infections are displayed in the supplemental material (Fig. S7). All growth curves are the means of 4 technical replicates and are representative of experiments conducted on different days and with different batches of urine. Values for competitive infections of arginine mutants versus the wild type are combinations of at least two biological replicates of 10 mice per replicate.

Several studies have suggested a role for arginine metabolism during UPEC UTI (37, 59–63). However, *argI* is the only *arg* gene under positive selection in UPEC. To further investigate the role of arginine metabolism and the function of ArgI during UTI, we generated individual strains with mutations in *argA* and *argG*, two genes predicted to encode metabolic enzymes that come biochemically before and after ArgI, respectively, in the arginine biosynthetic pathway (Fig. 3A and Fig. S6) (47, 64). These genes are each in monocistronic operons, and thus, their mutation is not predicted to induce polar effects. An Δ*argA* mutant should have arginine synthesis disrupted upstream of the substrate for ArgI, while an Δ*argI* mutant should have synthesis disrupted upstream of the substrate for ArgG (Fig. 3A and Fig. S6) (47, 64). In agreement with previous work in CFT073, all three *arg* mutants had similar phenotypes *in vitro*, exhibiting arginine auxotrophy in minimal defined media and a lower cell density at entry into stationary phase when grown in filtered pooled human urine (Fig. 3B and Fig. S6) (36). In particular, high-cell-density growth in urine was restored by supplementation of arginine or of the biosynthetic intermediates downstream of the mutated enzymatic step (Fig. 3C and Fig. S6). We then tested the *argA* and *argG* mutants in competitive chronic cystitis infections. Both the Δ*argA* and Δ*argG* mutants also had significant defects. In particular, the Δ*argG* mutant was similar to the Δ*argI* mutant, in that they were rapidly outcompeted by wild-type UTI89 in the urine samples [median log[CI] > 4.0 by 14 dpi] and had median log(CI) values from the bladders that were below −4.0 in mice at 28 dpi (Fig. 3D to F).

10.1128/mBio.02318-20.6 FIG S6 Chromosomal arrangement of the arginine synthesis genes in UTI89 and growth of arginine synthesis mutants. (A) Gene locations and direction taken from reference 47. (B) M9 is a minimal medium supplemented with glucose and ammonia. Urine was pooled, and filtered human urine was supplemented with 10 mM citrulline or ornithine as indicated. All growth curves are the means of 4 technical replicates and are representative of experiments conducted on different days and with different batches of urine. Download FIG S6, PDF file, 0.2 MB. Copyright © 2020 Hibbing et al. 2020 Hibbing et al. This content is distributed under the terms of the Creative Commons Attribution 4.0 International license.

10.1128/mBio.02318-20.7 FIG S7 Full arginine synthesis mutant CFU data and resolved-infection time course. Infection and competitive outcomes of 28-day infections with equal numbers of differentially marked wild-type and *arg* mutant UTI89 organisms. CFU graphs show the total bacteria recovered from the urine and tissues of the infections at the indicated time points. The cutoff indicates the level of persistent bacteriuria necessary for an infection to be classified as chronic cystitis. Resolved CI graphs show the log_10_(CI_(mutant/wild type)_) from mice with at least one incidence of low-titer bacteriuria (<10^4^ CFU/ml urine). Download FIG S7, PDF file, 0.3 MB. Copyright © 2020 Hibbing et al. 2020 Hibbing et al. This content is distributed under the terms of the Creative Commons Attribution 4.0 International license.

### B2 strains of E. coli have a divergent allele of *argI*.

**FIG 4:**
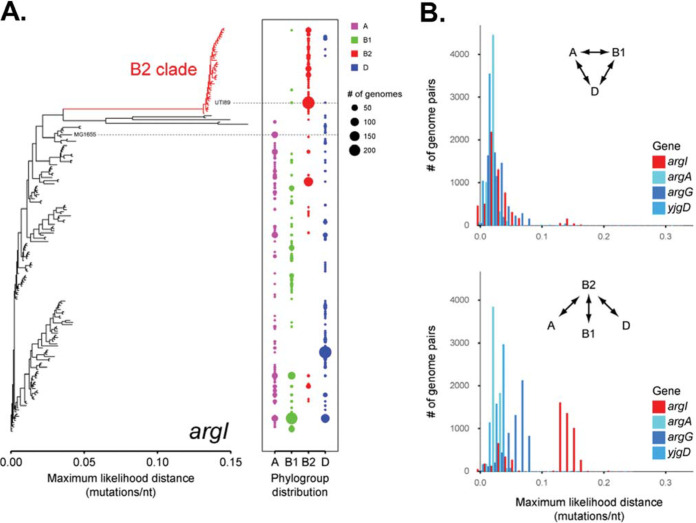
B2 alleles of *argI* are distinct. (A) Approximate maximum-likelihood tree of unique *argI* alleles. The *x* axis indicates mutations per nucleotide (nt). Multiple strains may have the same *argI* allele, and strains from different phylogroups may share the same allele. The phylogroup breakdown for each *argI* allele is shown by colored circles at the right, with the size of the circle representing the number of strains and the color indicating the phylogroup of those strains, as shown in the legend. The red branch of the *argI* tree on the left indicates a part of the tree that contains *argI* alleles that are mostly from B2 strains. Alleles found in UTI89 and MG1655 are indicated. (B) Histograms of sequence divergence between *argI* alleles from different phylogroups. Random pairs of strains (the two strains in each pair were from different phylogroups) were selected, and the maximum-likelihood distance between their *argI* genes was calculated. A total of 2,000 strain pairs for each phylogroup comparison were chosen, and the histogram of distances is plotted. The same process was repeated for different genes, as indicated by the colors of the bars. The top graph only plots distances for pairs of strains in which neither strain was a B2 strain. The bottom graph plots distances for pairs of strains where one of the strains was a B2 strain.

The above-described experiments with the Δ*argA* and Δ*argG* mutants argue that the role of ArgI in arginine biosynthesis does not explain why it is evolving under positive selection. To understand whether allelic variation (as opposed to deletion mutants) might provide further insights (as it did with *fimH*), we assessed the conservation of the *arg* genes via a pairwise comparison of arginine anabolic protein sequences from UTI89 and the commensal E. coli isolate MC4100 (Fig. S8). In this comparison, the ArgI sequences had 20 variant residues out of 334 total amino acids (94% identity), while the other arginine anabolic proteins were 98 to 100% identical between these isolates (Fig. S8). Interestingly, the majority of the variable amino acids were spatially far from both the enzymatic active site and the trimer interfaces (Fig. S8). We then examined the same *arg* genes in a set of 2,274 publicly available E. coli genomes (all NCBI RefSeq assemblies as of 26 April 2016). Again, *argI* was unique in that there was a largely B2-specific clade of *argI* alleles which were separated from the other E. coli *argI* alleles by a relatively long evolutionary branch (Fig. 4A). While B2-specific alleles were found in other *arg* genes, they were not as clearly divergent from the other E. coli alleles. (Fig. 4B). The conserved *argI* allele in the B2 clade of UPEC was significantly divergent (lowest nucleotide and predicted amino acid identity) from *argI* alleles in the other E. coli clades. This demonstrates that there is a conserved *argI* allele in the B2 clade, which was divergent from *argI* in the other B2 clades. (Note that while sequence differences are required for positive-selection detection algorithms to work, the existence of positive selection does not necessarily correlate with sequence diversity in a given gene.)

10.1128/mBio.02318-20.8 FIG S8 Arginine synthesis protein variation between UTI89 and MC4100 and impacts of allelic variation on growth in urine. (A) Arginine synthesis protein amino acid identity and similarity between UTI89 and MC4100. (B) Amino acid alignment between ArgI_UTI89_ and ArgI_MC4100_. Variable residues are highlighted in pink. (C) Top and bottom views of the crystal structure of the ArgI homotrimer modified from PDB entry 2OTC, represented in the ribbon diagrams. Each individual monomer is indicated by a different color (orange, yellow, and pink). Active-site residues are indicated by blue spheres, and residues that vary between ArgI_UTI89_ and ArgI_MC4100_ are indicated by red spheres (Y. Ha, M. T. McCann, M. Tuchman, and N. M. Allewell, Proc Natl Acad Sci U S A 94:9550–9555, 1997; L. B. Murata and H. K. Schachman Protein Sci 5:709–718, 1996). (D) Growth of otherwise isogenic UTI89 strains in pooled, filtered human urine that have *argI* deleted or replaced with the UTI89 *argI* allele (U) or MC4100 *argI* allele (M) and expressing a kanamycin resistance gene (Km) or a chloramphenicol resistance gene (Cm) integrated into the HK site compared to wild-type UTI89. Curves are means of 4 technical replicates. (E) Competitive growth of UTI89 expressing the UTI89 allele of *argI* versus UTI89 expressing the MC4100 allele of *argI* in pooled, filtered human urine. Download FIG S8, PDF file, 0.6 MB. Copyright © 2020 Hibbing et al. 2020 Hibbing et al. This content is distributed under the terms of the Creative Commons Attribution 4.0 International license.

### A B2 allele of *argI* has an additional *in vivo* function during competitive chronic UTI.

To assess whether *argI* has an additional role during UTI aside from arginine biosynthesis, we generated allelic replacement strains of UTI89, reintegrating the UTI89 (a B2 strain) or MC4100 (a non-B2 strain) allele of *argI* (designated *argI*_UTI89_ and *argI*_MC4100_, respectively) into the original *argI* locus in UTI89 using a scarless cloning technique (65). As expected, both *argI* alleles complemented all *in vitro* growth phenotypes (arginine auxotrophy in minimal media and low-density transition to stationary phase in urine) (Fig. S8). To further test for subtle fitness defects, we performed competitive *in vitro* growth assays between strains carrying either allele; again, no differences were detectable between otherwise isogenic strains carrying these two *argI* alleles (Fig. S8).

**FIG 5:**
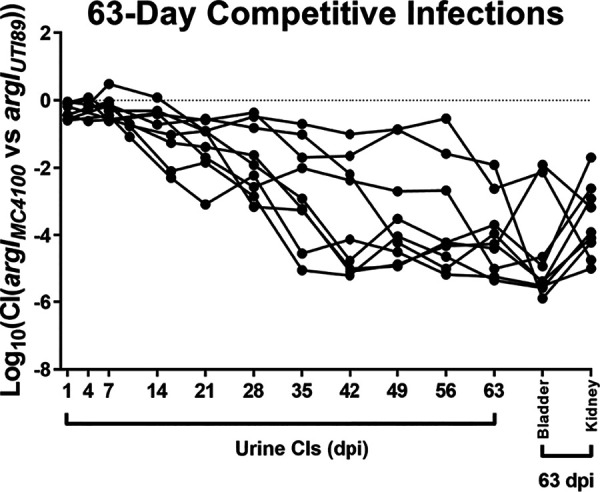
The UTI89 variant of ArgI confers a competitive advantage over the MC4100 variant during chronic cystitis. The competitive infection defect, indicated by log_10_(CI), of UTI89 carrying the *argI*_MC4100_ relative to UTI89 carrying the *argI*_UTI89_ allele reintegrated into the chromosomal location of the original copy of *argI* in the UTI89 genome in the urine during and in the bladder and kidney tissue at sacrifice after 63-day coinfections is shown. Only mice with persistent bacteriuria of >10^4^ CFU/ml were included in the analysis. Median bladder log_10_(CI)s are significantly different from a hypothetical value of 0 by the Wilcoxon signed-rank test with *P* values of <0.05. Antimicrobial resistance markers were swapped to ensure that the resistance markers did not contribute to the phenotype, and the raw CFU data of all the infections and the log_10_(CI) values of the resolved infections are displayed in the supplemental material (Fig. S9). Each combination of *argI* allele and antimicrobial marker was assessed with a single biological replicate of 10 mice each, resulting in two biological replicates comparing UTI89 *argI*_UTI89_ versus UTI89 *argI*_MC4100_.

We then performed competitive, *in vivo* chronic infections between these strains carrying the *argI*_UTI89_ and *argI*_MC4100_ alleles. These infections were performed as described above, except they were allowed to proceed longer (63 dpi), as an initial pilot experiment suggested that the full effect of the non-B2 *argI* allele had not manifested by 28 dpi (data not shown). Remarkably, despite no detectable differences *in vitro*, bacteria carrying *argI*_UTI89_ outcompeted those with the *argI*_MC4100_ allele in the urine, bladders, and kidneys of chronically infected mice, reaching a median log_10_(CI) of −5.4 in the bladder at sacrifice at 63 dpi (Fig. 5 and Fig. S9). The strain carrying *argI*_MC4100_ was nearly undetectable at this time. Importantly, at 28 dpi (matching the time point for previous infections), the *argI*_MC4100_ strain was already significantly outcompeted by the *argI*_UTI89_ strain in urine (Fig. 5).

10.1128/mBio.02318-20.9 FIG S9 Full *argI* allele CFU data and antimicrobial resistance allele swap. Infection and competitive outcomes of 63-day infections with equal numbers of differentially marked UTI89 isolates expressing different alleles of *argI*. CFU graphs show the total bacteria recovered from the urine and tissues of the infections at the indicated time points. The cutoff indicates the level of persistent bacteriuria necessary for an infection to be classified as chronic cystitis. Chronic CI graphs show the log_10_(CI_(ArgI_MC4100___/ArgI_UTI89___)_) from mice with persistent high titer bacteriuria (>10^4^ CFU/ml urine). Resolved CI graphs show the log_10_(CI_(ArgI_MC4100___/ArgI_UTI89___)_) from mice with at least one incidence of low-titer bacteriuria (<10^4^ CFU/ml urine). The top 3 graphs show competitions of UTI89 expressing the UTI89 allele of *argI* and a kanamycin resistance gene against UTI89 expressing the MC4100 allele of *argI* and a chloramphenicol resistance gene. The bottom 3 graphs show competitions of UTI89 expressing the UTI89 allele of *argI* and a chloramphenicol resistance gene against UTI89 expressing the MC4100 allele of *argI* and a kanamycin resistance gene. The chronic CI data were combined to make Fig. 5. Download FIG S9, PDF file, 0.3 MB. Copyright © 2020 Hibbing et al. 2020 Hibbing et al. This content is distributed under the terms of the Creative Commons Attribution 4.0 International license.

## DISCUSSION

Different E. coli strains are known to differ in their propensity to cause disease in humans (and other organisms), giving rise to disease syndrome-based terms such as UPEC, enterohemorrhagic E. coli (EHEC), enteropathogenic E. coli (EPEC), avian pathogenic E. coli (APEC), and neonatal meningitis-causing E. coli (NMEC), among others. In other words, some E. coli strains are better adapted to survive in different habitats. A firm genetic definition of these various types of pathogenic E. coli remains elusive; however, most of the specific disease phenotypes have been attributed to known or putative virulence factors, although many of these have not been fully experimentally verified. UPEC, in particular, is a heterogeneous group of E. coli strains; while most are found within the B2 and D clades, there remains substantial variation in gene content even among B2 clade UPEC strains and no clear genetic signature of UPEC (66, 67). One approach to identifying genetic adaptations that enable strains to cause urinary tract infections (or other diseases) is to look for loci under positive selection (47, 68).

Positive selection is an evolutionary process whereby organisms with genetic changes that increase fitness are more likely to survive or reproduce, thus increasing the frequency of those changes in the population with time. In the context of virulence, the genetic factors that enable some strains to cause a specific disease are those that increase fitness and therefore should be evolving under positive selection. In the specific case of UPEC, a direct connection between positive selection and virulence-related genes has been posited (47, 68). One of the best-characterized examples in UPEC is the *fimH* gene, which encodes the FimH tip adhesin that tips the type 1 pilus. FimH has a critical role in the establishment of UTI as the primary binding determinant for UPEC to specifically attach to and invade bladder epithelial cells by engaging mannosylated surface proteins (17, 20, 69). The *fimH* gene is evolving under positive selection (43, 46, 48, 68). Interestingly, the residues detected as increasing fitness initially had no detectable *in vitro* role in binding to mannose (48). Subsequent work showed that the amino acids under positive selection in FimH affected the dynamics of switching between high- and low-affinity mannose binding states, which was a previously unappreciated step during UTI (49, 50).

In this study, we comprehensively tested a set of 29 additional PSGs that were previously identified by comparing sequences of UPEC versus non-UPEC E. coli strains. Deletion of 8 of these genes lowered fitness in a mouse model of chronic UTI. This model mimics persistent infections experienced by some human patients. In this model, high titers of UPEC are continuously present in the urine for the lifetime of the animal or until the infection is cleared by antibiotics, implying that there may be a strong selection for utilization of available nutrients (10, 12, 13). Interestingly, PSG mutants demonstrated infection defects only when competing with an otherwise isogenic wild-type strain. In humans, UTIs likely arise from the introduction of an inoculum consisting of mixed bacterial species and E. coli strains from gastrointestinal and vaginal reservoirs into the urinary tract (70–73). At the time of diagnosis, however, UTIs are generally clonal infections consisting of high bacterial loads of a single strain (74–76), implying that naturally occurring human UTIs may, in fact, also be competitive infections. This competition will occur between E. coli and other bacteria in the inoculum, which should include other members of the vaginal or perineal flora (such as *Lactobacillus*); however, multiple strains of individual species (particularly E. coli) could also be present in the inoculum, either closely or distantly related to one another, mimicking the competitive experimental infections we have studied here.

The importance of growth and arginine metabolism led us to perform further studies on *argI* (37, 59–63). Mutation of *argI* abolishes the ability of UPEC to synthesize arginine, leading to auxotrophy for arginine. To test the hypothesis that arginine metabolism is important in chronic UTI, we made deletion mutations in two other genes, *argA* and *argG*, which also result in arginine auxotrophy. As expected, these mutations also led to a fitness defect in chronic UTI. These results demonstrate that amino acids, in particular arginine, are present at low levels in urine and that the ability to synthesize them *de novo* confers a competitive advantage to UPEC during UTI, but they do not explain why *argI* is the only arginine synthesis gene evolving under positive selection.

We therefore reexamined the sequence of *argI* and other arginine metabolic genes in a broader set of >2,200 E. coli genome sequences, which indicated, consistent with previous reports, that *argI* is evolving under positive selection and has specifically diverged in B2 strains of E. coli. This divergence has a specific functional consequence *in vivo*, as a UPEC strain carrying either a B2 allele or an A allele (a clade of E. coli containing many commensal GI strains) has no auxotrophic phenotype for arginine; however, the strain carrying the B2 allele outcompetes an isogenic strain carrying the A allele as if the latter were an *argI* knockout. Interestingly, the clade D *argI* allele of E. coli was not under positive selection, despite the fact that E. coli strains from this clade are frequently observed causative agents of UTI (39). This finding suggests that there may be multiple solutions that E. coli has evolved to facilitate persistence in the urinary tract. We suggest that further positive selection analysis focusing on clade D E. coli might reveal aspects of such an alterative mechanism.

These results with *argI* form a strong parallel with those previously observed for *fimH*. Both genes have a clear function that is readily measured *in vitro* and known to be important for UTI *in vivo*. In addition, both genes have allelic variants naturally found among E. coli that do not obviously affect their function *in vitro* but result in strong fitness defects *in vivo*. Similarly, it was recently shown that the oxidative fumarase FumC is essential for fitness during UTI pathogenesis, but this essentiality appears to be predominantly associated with the role FumC plays in coping with iron limitation, via its ability to function enzymatically without an Fe-S cluster, rather than its specific role in the tricarboxylic acid (TCA) cycle (77). We therefore hypothesize that, like with FimH and FumC, a potential second, and possibly more subtle, function not currently appreciated *in vitro* exists for ArgI. This second ArgI function would have specifically evolved in B2, and its importance during chronic UTI would account for its being under positive selection (though other environments or disease states may select for it as well). The arginine synthesis step catalyzed by *argI* is situated at a branch point where biosynthetic precursors can be shunted from arginine synthesis into the biosynthetic pathway for several polyamines. The flux through the synthesis pathways of arginine and of polyamines, which are multifunctional compounds that have been shown to contribute to stress responses in UPEC, represent one potential mechanism by which the B2 allele of *argI* contributes to fitness (61, 78, 79). Conversely, the enzymatic steps catalyzed by ArgA and ArgG are relatively distal from this branch point and would likely have less impact on the relative levels of arginine, ornithine, and the other arginine-derived polyamines, suggesting one possible reason why *argI* is under positive selection in clade B2 E. coli but *argA* and *argG* are not.

We therefore conclude that positive selection analysis is indeed a powerful and complementary technique for identifying genes important for virulence. The success rate of PSG validation (deletion of 10/29 PSGs has a fitness defect, during chronic infection [8 PSGs] and/or QIR formation [6 PSGs]) compares favorably to other methods for identifying UPEC virulence genes, such as gene presence/absence analysis, candidate gene analysis (mostly targeted at “traditional” virulence factors or pathogenicity islands), and transcriptomics analyses (38, 40, 80–84). Of note, other mutant screens, such as Tn-seq, have very high validation rates. This is likely due to the fact that the “putative virulence factors” identified in the initial large-scale mutagenesis Tn-seq library screens are most often validated using the same pathogenicity model under which they were first selected. PSG analysis is more similar to a bioinformatic genomic comparison to determine presence/absence of genes in particular pathogenic strains compared to nonpathogenic strains. These putative virulence genes and/or specific gene alleles determined bioinformatically may be selected for in any of a number of different environments or conditions under which these strains reside and/or cause infection. Thus, the success of validating the role of any genomically identified candidate virulence factors is dependent upon which model they are tested in. We tested our PSGs in a UTI model and found that strains with deletions in any of 10 of them had measurable UTI phenotypes. It may be possible that the other 19 PSGs for which we found no UTI phenotype may provide a selective advantage in other habitats related to UTI conditions, residence in the gastrointestinal tract (GIT), or vaginal reservoirs (85, 86). Further, the particular alleles of PSGs may have phenotypes that cannot be elucidated by testing deletion mutants but may require testing using allele swaps.

In addition, as shown for *fimH* and now *argI*, positive selection analysis potentially captures alternative gene functions that are specifically required *in vivo*. More generally, positive selection analysis is applicable to any infectious agent, sequences of which are available at exponentially increasing rates. In particular, positive selection may be especially useful for studying pathogenic processes that, like UTI, have stringent bottlenecks and strong founder effects, such as those of HIV, Salmonella enterica serovar Typhimurium, and Borrelia burgdorferi (87–90).

## MATERIALS AND METHODS

### Ethics statement.

All animal experiments were conducted in accordance with the National Institutes of Health guidelines for the housing and care of laboratory animals as well as institutional regulations after pertinent review and approval by the Animal Studies Committee at the Washington University School of Medicine (protocol number 21080276). Human urine used for growth determination was collected from healthy consenting volunteers according to Hultgren lab human studies protocol number 201207143.

### Media, reagents, and mutant generation.

The UPEC strains used in this study are all derivatives of the human cystitis isolate UTI89 and are listed in Table S1 (17, 91). In general, bacteria were grown and propagated in Luria-Bertani (LB) broth and plated for isolation on LB agar (BD). Where necessary, the medium was supplemented with 50 μg/ml kanamycin, 50 μg/ml spectinomycin, 100 μg/ml ampicillin, and/or 1 mM isopropyl β-d-1-thiogalactopyranoside (Gold-Bio). Human urine was collected from at least 2 healthy volunteers and filter sterilized through a 0.22-μm filter (Millipore). Biological replicates of growth curves were conducted with different batches of urine collected on different days. Where indicated, urine was supplemented with 10 mM ornithine, citrulline, or arginine (Sigma-Aldrich).

All deletion mutants were generated in the prototypical cystitis isolate UTI89 using the λRed recombinase system (92, 93). The allelic replacement of *argI* was performed using a previously described, λRed recombinase-based negative-selection system (65). To facilitate *in vivo* competition assays, kanamycin or chloramphenicol resistance markers were inserted into the HK site using the standard λRed recombinase protocol (49, 94). All deletion and allelic replacement mutants were confirmed by Sanger sequencing.

### Growth curves.

Growth curves were acquired on a Tecan infinite M200 Pro (Tecan). Cultures were diluted 1:1,000 into 200 μl of the appropriate medium with arginine, citrulline, or ornithine supplementation where appropriate in CellStar clear polystyrene 96-well flat-bottom plates (Greiner Bio-One) and incubated at 37°C with shaking for 10 to 15 h. Each curve is the average of 4 technical replicates, and the curves are representative of at least 3 independent experiments.

### Hemagglutination.

Hemagglutination assays were performed as described previously (95, 96).

### Mouse models of acute and chronic UTI.

Female, 6- to 7-week-old C3H/HeN HSD mice were acquired from Envigo Laboratories and allowed to acclimate to the Washington University Medical School animal housing facility for 1 week. General infections were performed transurethrally as described previously (11). For single-strain infections, the inoculum contained 2 × 10^7^ to 4 × 10^7^ CFU of a derivative of UTI89 containing a chromosomally integrated kanamycin resistance gene either at the pathogenically neutral lambda *attP* site (94) or at the site of the desired PSG or arginine synthesis gene. Competitive infections were inoculated with 2 × 10^7^ to 4 × 10^7^ CFU each of a derivative of UTI89 containing a chromosomally integrated spectinomycin resistance gene at the pathogenically neutral HK site (16) and one of the kanamycin resistance (Km^r^) gene-containing strains mentioned above, including the wild type to control for pleotropic effects of the kanamycin and spectinomycin resistance genes on fitness during pathogenesis. Allele swap experiments assessing the impact *argI* variation were performed with UTI89 *argI*_UTI89_ + Km^r^ in the HK site competing against UTI89 *argI*_MC4100_ + Cm^r^ (chloramphenicol resistance gene) in the HK site and with UTI89 *argI*_UTI89_ + Cm^r^ in the HK site competing against UTI89 *argI*_MC4100_ + Km^r^ in the HK site.

UT tissue burdens were determined as described previously (11). For long-term infections, bacteriuria was monitored as described previously at various time points from 1 to 63 dpi (11). Where appropriate, competitive indices were determined as log_10_[(Spec^r^ CFU/Kan^r^ CFU)/inoculum (Spec^r^ CFU/Kan^r^ CFU)] or log_10_[(*argI*_MC4100_ CFU/*argI*_UTI89_ CFU)/inoculum (*argI*_MC4100_ CFU/*argI*_UTI89_ CFU)] to control for variations in the initial inoculum.

### Phylogenetic analysis of arginine anabolism.

All RefSeq assemblies listed for Escherichia coli (as of 26 April 2016) were downloaded from NCBI GenBank. For a given gene, orthologs were identified using TBLASTN (97); the UTI89 allele of that gene was used as the query against each of the individual assembled genomes. For each genome, only the best single hit that was at least 90% identical over at least 90% of the total length of the UTI89 allele was kept. Unique alleles (based on DNA sequence) were aligned as translated protein sequences; then this protein alignment was imposed on the DNA sequences. Approximate maximum-likelihood trees were created using FastTree 2 (with the –nt and –gtr command line switches) (98). Phylogroups were assigned using an *in silico* implementation of a triplex PCR (99). All analyses utilized custom Perl scripts. Visualization was done in R (version 3.2.2; https://www.R-project.org) using the ggtree (100), ape (101), and ggplot2 (102) packages.
