# Supplementary material for: Adaptation of Arginine Synthesis among Uropathogenic Branches of the Escherichia coli Phylogeny Reveals Adjustment to the Urinary Tract Habitat
Source: mBio. 2020 Sep 29;11(5):e02318-20. doi: 10.1128/mBio.02318-20 (PMC7527732; doi:10.1128/mBio.02318-20)
Supplement: FIG S1 [file mBio.02318-20-sf001.pdf]

Figure S1i.

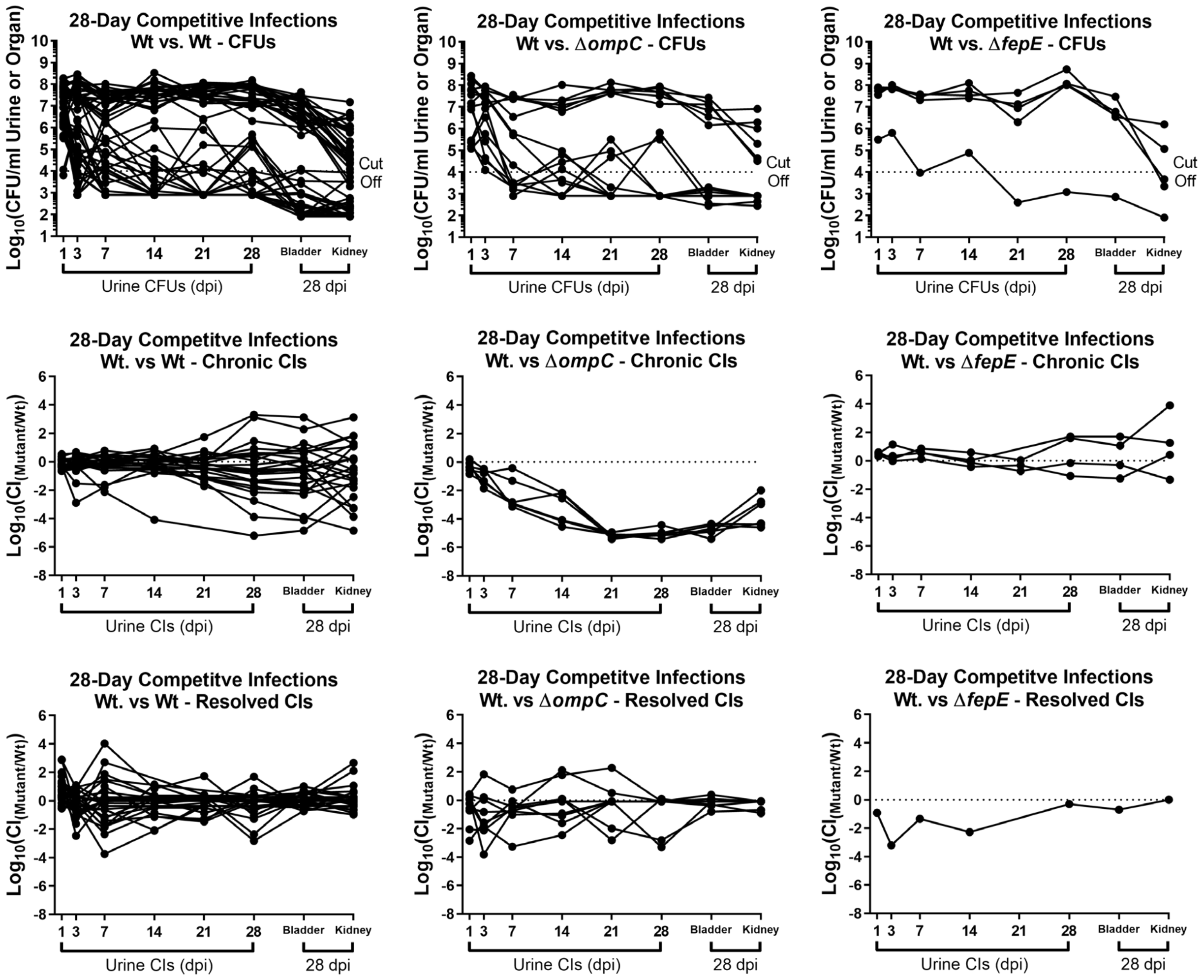

28-Day Competitive Infections
Wt vs. Wt - CFUs
Wt vs. ΔompC - CFUs
Wt vs. ΔfepE - CFUs
Log10(CFU/ml Urine or Organ)
Cut Off
Urine CFUs (dpi)
28 dpi
Bladder
Kidney
28-Day Competitve Infections
Wt. vs Wt - Chronic CIs
Wt. vs ΔompC - Chronic CIs
Wt. vs ΔfepE - Chronic CIs
Log10(CI(Mutant/Wt))
Urine CIs (dpi)
Wt. vs Wt - Resolved CIs
Wt. vs ΔompC - Resolved CIs
Wt. vs ΔfepE - Resolved CIs

Figure S1ii.

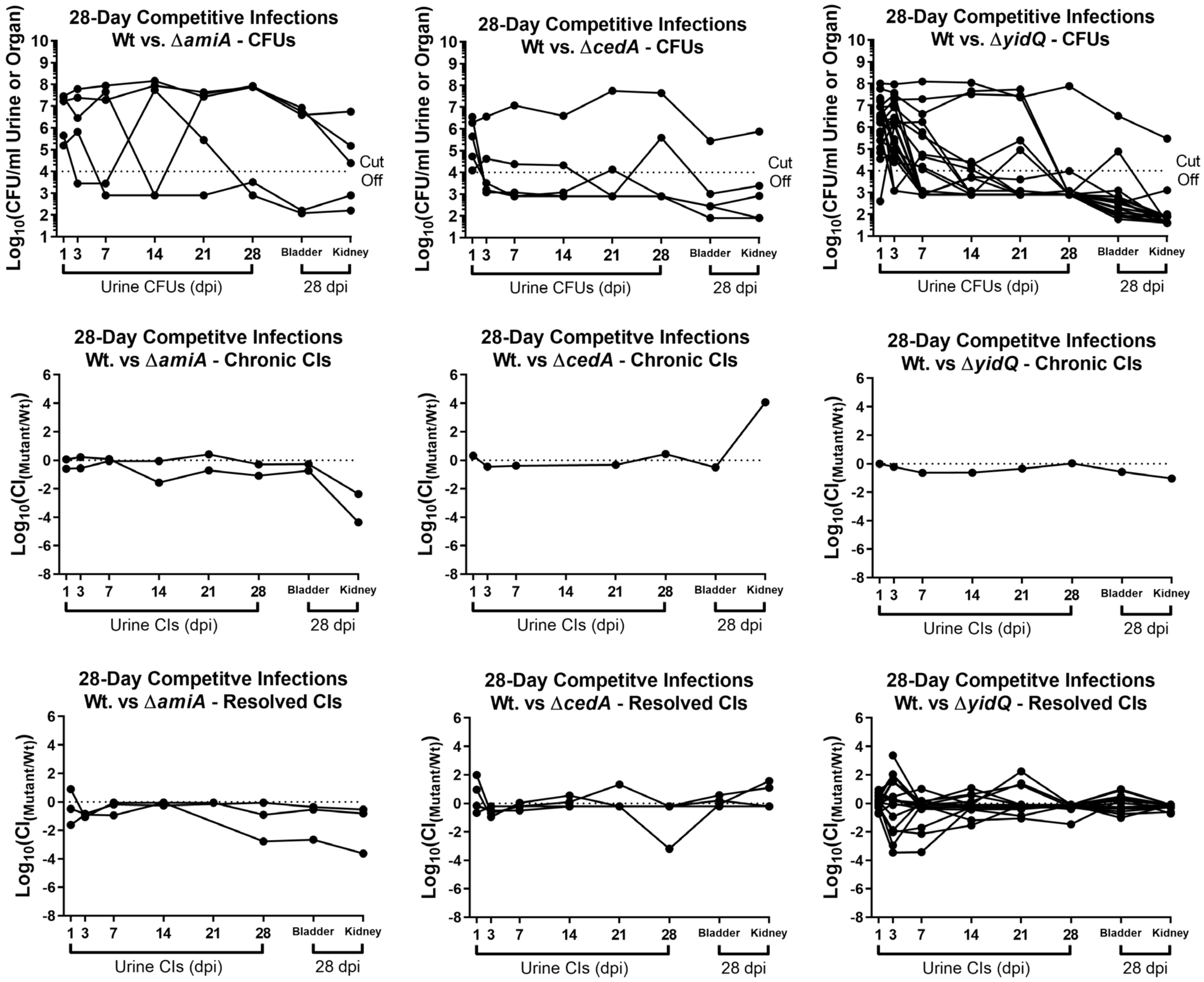
28-Day Competitive Infections
Wt vs. ΔamiA - CFUs
28-Day Competitive Infections
Wt vs. ΔcedA - CFUs
28-Day Competitive Infections
Wt vs. ΔyidQ - CFUs
Log10(CFU/ml Urine or Organ)
Cut Off
Bladder
Kidney
Urine CFUs (dpi)
28 dpi
28-Day Competitve Infections
Wt. vs ΔamiA - Chronic CIs
28-Day Competitve Infections
Wt. vs ΔcedA - Chronic CIs
28-Day Competitve Infections
Wt. vs ΔyidQ - Chronic CIs
Log10(CI(Mutant/Wt))
Urine CIs (dpi)
28-Day Competitve Infections
Wt. vs ΔamiA - Resolved CIs
28-Day Competitve Infections
Wt. vs ΔcedA - Resolved CIs
28-Day Competitve Infections
Wt. vs ΔyidQ - Resolved CIs

Figure S1iii.

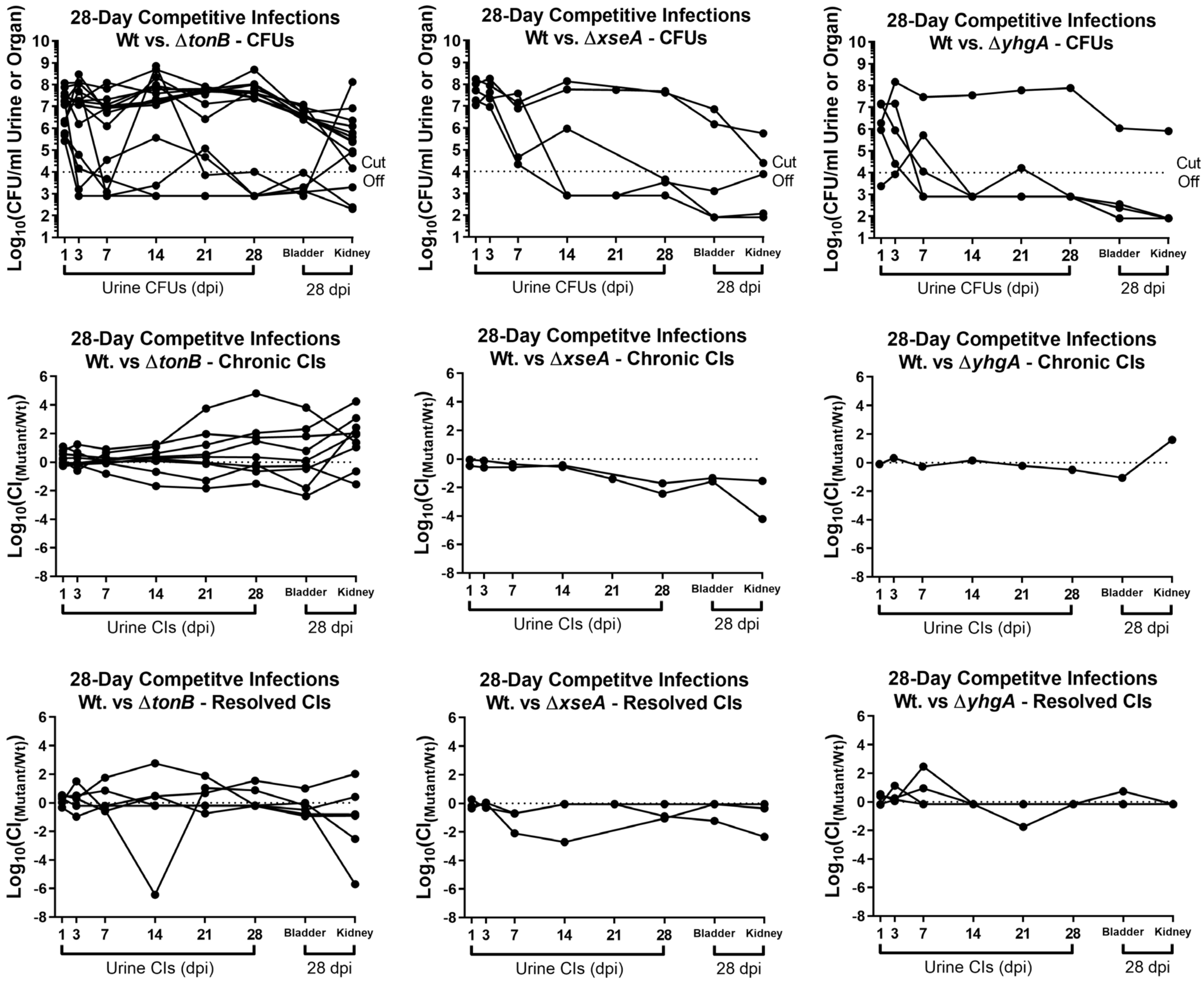

Figure S1iv.

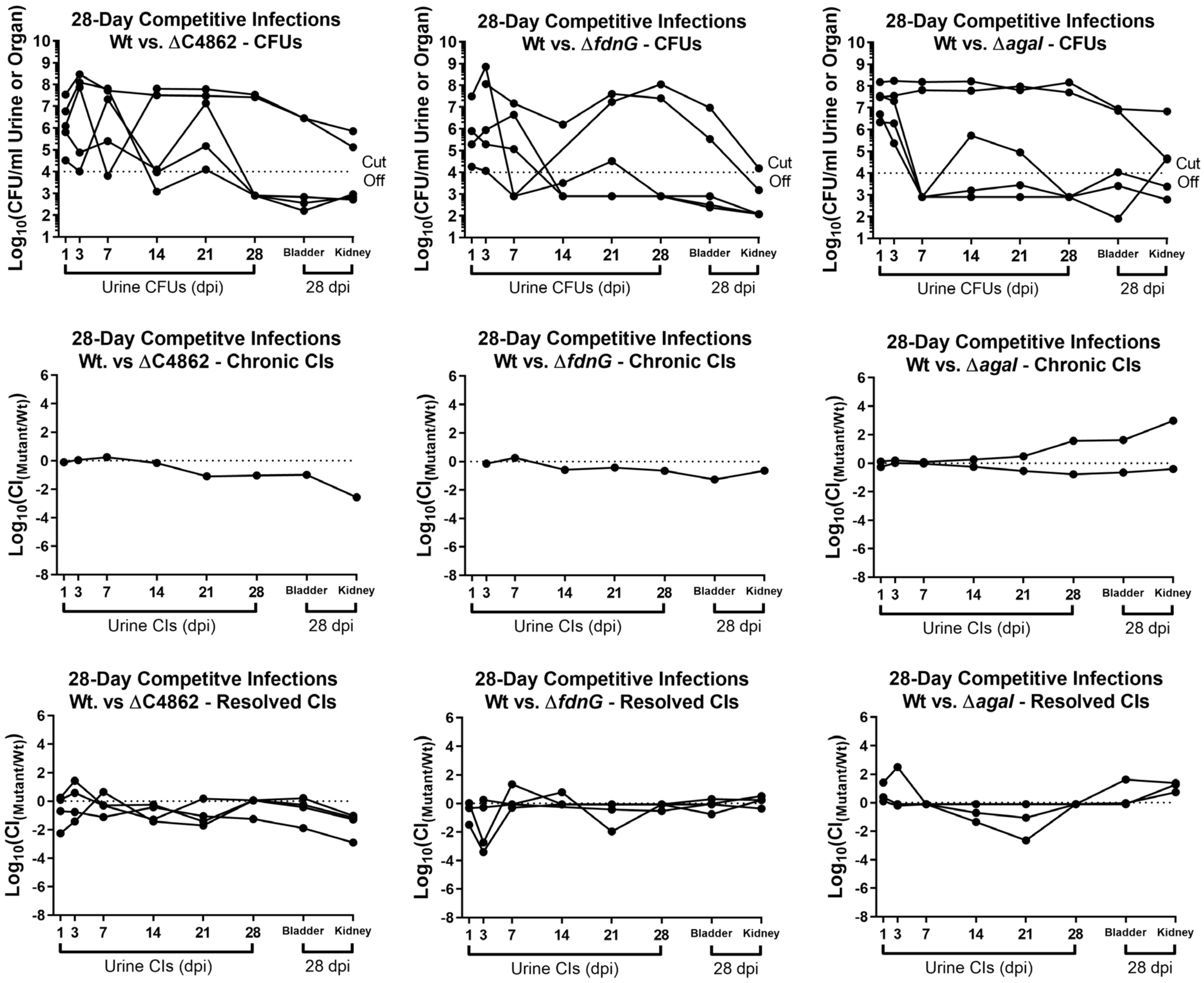

28-Day Competitive Infections
Wt vs. ΔC4862 - CFUs
28-Day Competitive Infections
Wt vs. ΔfdnG - CFUs
28-Day Competitive Infections
Wt vs. Δagal - CFUs
Log10(CFU/ml Urine or Organ)
Cut Off
Urine CFUs (dpi)
28 dpi
Bladder
Kidney
28-Day Competitve Infections
Wt. vs ΔC4862 - Chronic CIs
28-Day Competitive Infections
Wt vs. ΔfdnG - Chronic CIs
28-Day Competitive Infections
Wt vs. Δagal - Chronic CIs
Log10(CI(Mutant/Wt))
Urine CIs (dpi)
28 dpi
28-Day Competitve Infections
Wt. vs ΔC4862 - Resolved CIs
28-Day Competitive Infections
Wt vs. ΔfdnG - Resolved CIs
28-Day Competitive Infections
Wt vs. Δagal - Resolved CIs
Log10(CI(Mutant/Wt))
Urine CIs (dpi)
28 dpi

Figure S1v.

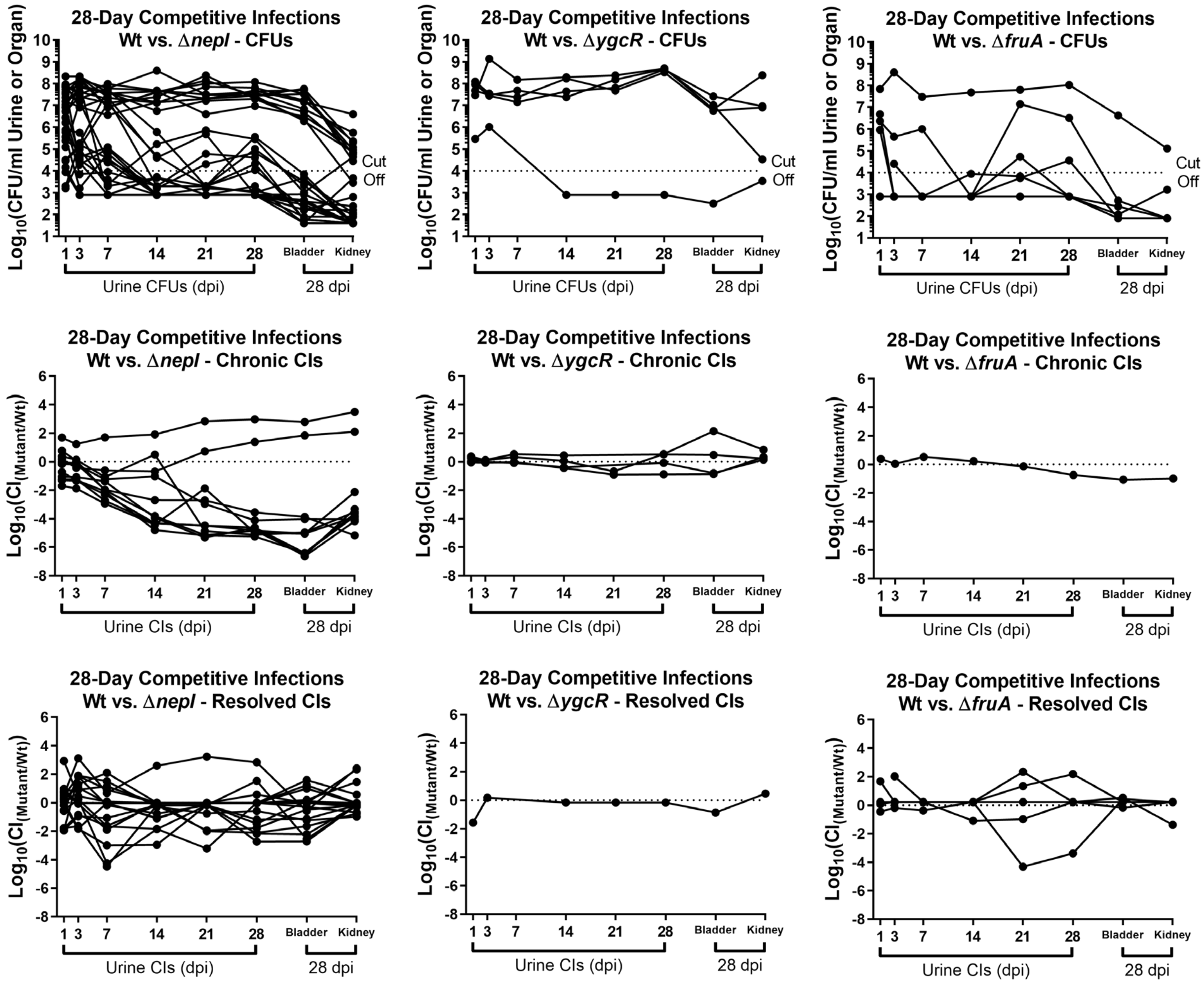
28-Day Competitive Infections
Wt vs. ΔnepI - CFUs
Log10(CFU/ml Urine or Organ)
Cut Off
Urine CFUs (dpi)
28 dpi
Bladder
Kidney
28-Day Competitive Infections
Wt vs. ΔygcR - CFUs
28-Day Competitive Infections
Wt vs. ΔfruA - CFUs
28-Day Competitive Infections
Wt vs. ΔnepI - Chronic CIs
Log10(CI(Mutant/Wt))
Urine CIs (dpi)
28-Day Competitive Infections
Wt vs. ΔygcR - Chronic CIs
28-Day Competitive Infections
Wt vs. ΔfruA - Chronic CIs
28-Day Competitive Infections
Wt vs. ΔnepI - Resolved CIs
28-Day Competitive Infections
Wt vs. ΔygcR - Resolved CIs
28-Day Competitive Infections
Wt vs. ΔfruA - Resolved CIs

Figure S1vi.

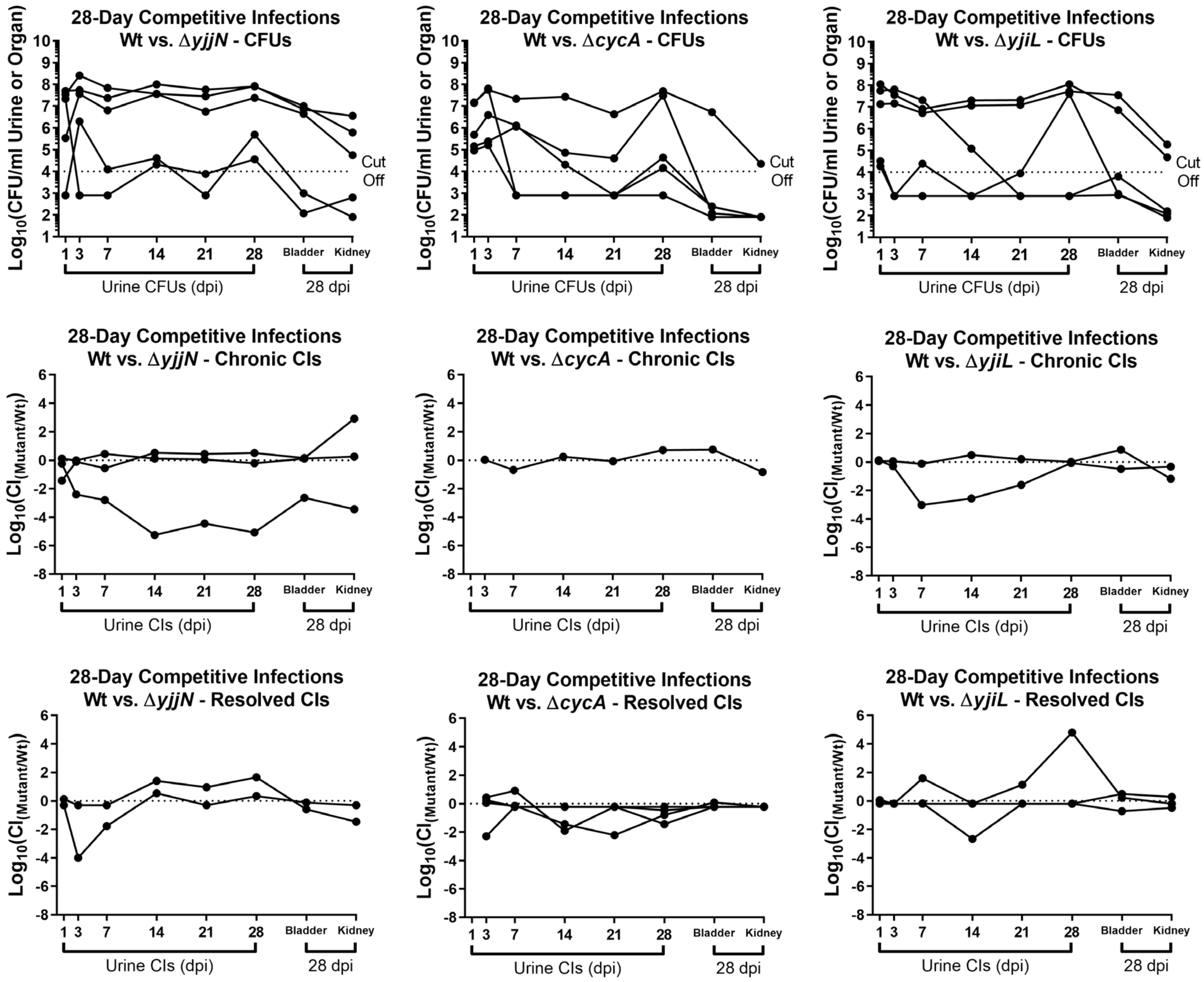
28-Day Competitive Infections
Wt vs. ΔyjjN - CFUs
Wt vs. ΔcycA - CFUs
Wt vs. ΔyjiL - CFUs
Wt vs. ΔyjjN - Chronic CIs
Wt vs. ΔcycA - Chronic CIs
Wt vs. ΔyjiL - Chronic CIs
Wt vs. ΔyjjN - Resolved CIs
Wt vs. ΔcycA - Resolved CIs
Wt vs. ΔyjiL - Resolved CIs
Log10(CFU/ml Urine or Organ)
Log10(CI(Mutant/Wt))
Cut Off
Bladder
Kidney
Urine CFUs (dpi)
Urine CIs (dpi)
28 dpi

Figure S1vii.

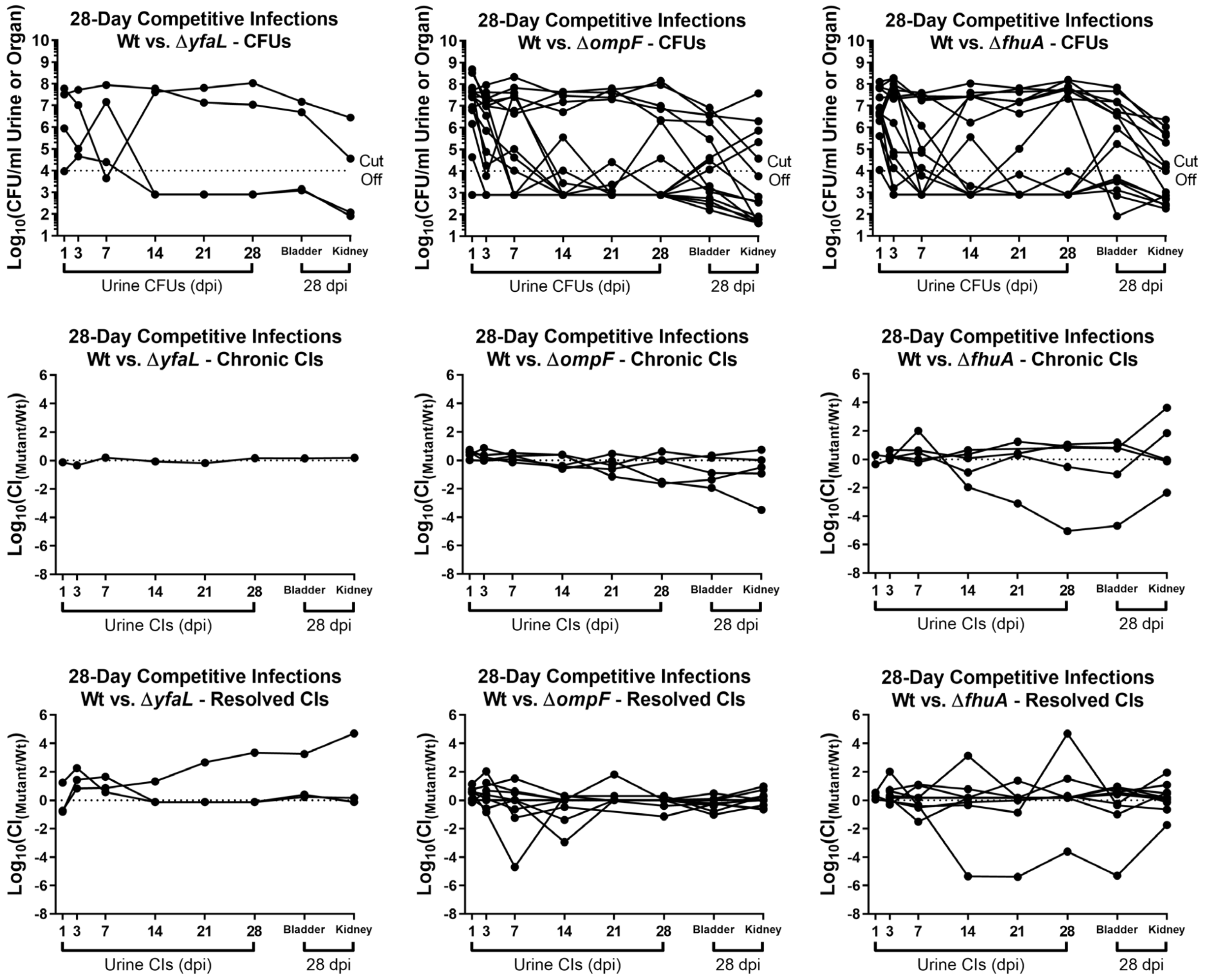

Figure S1viii.

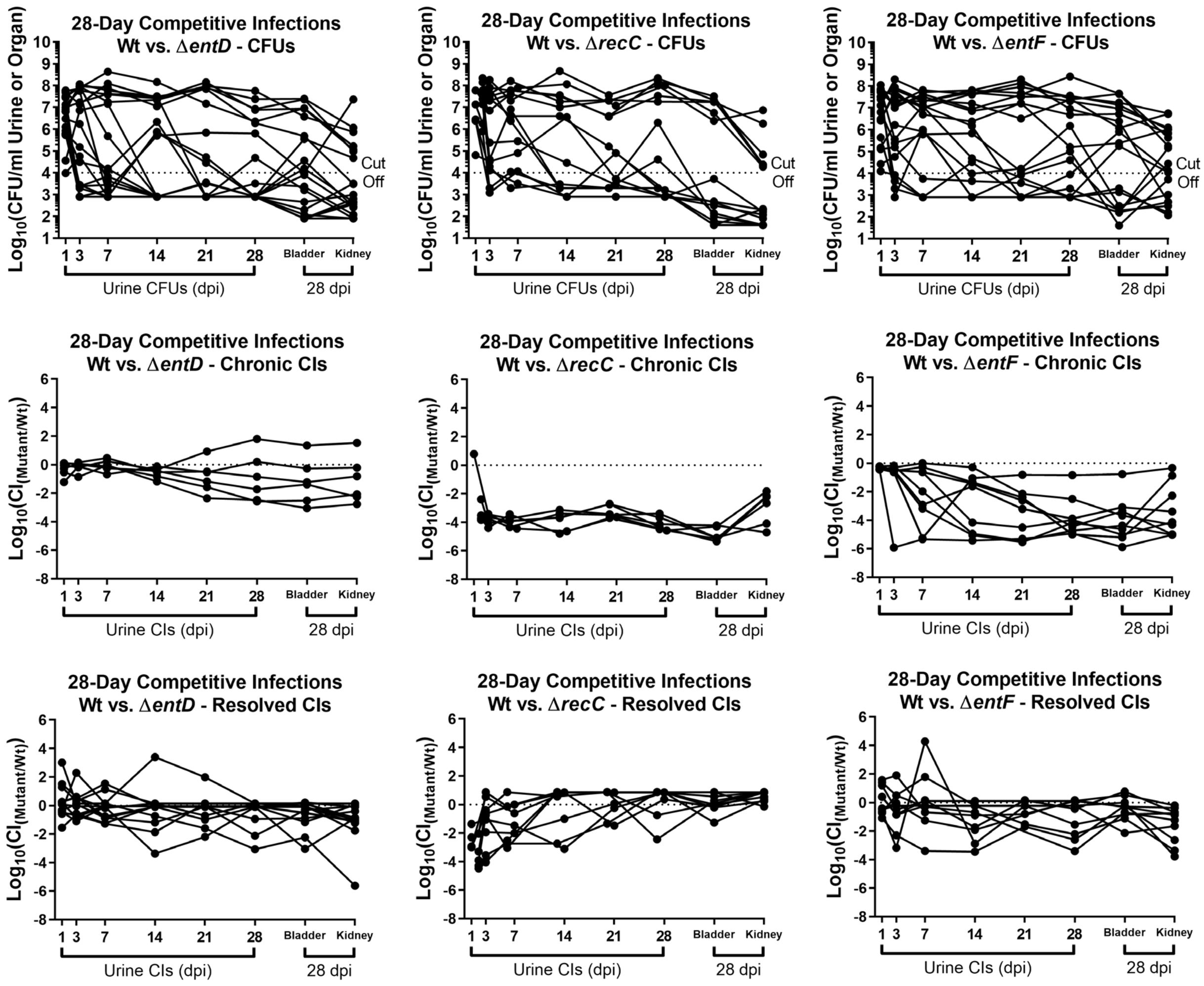

Figure S1ix.

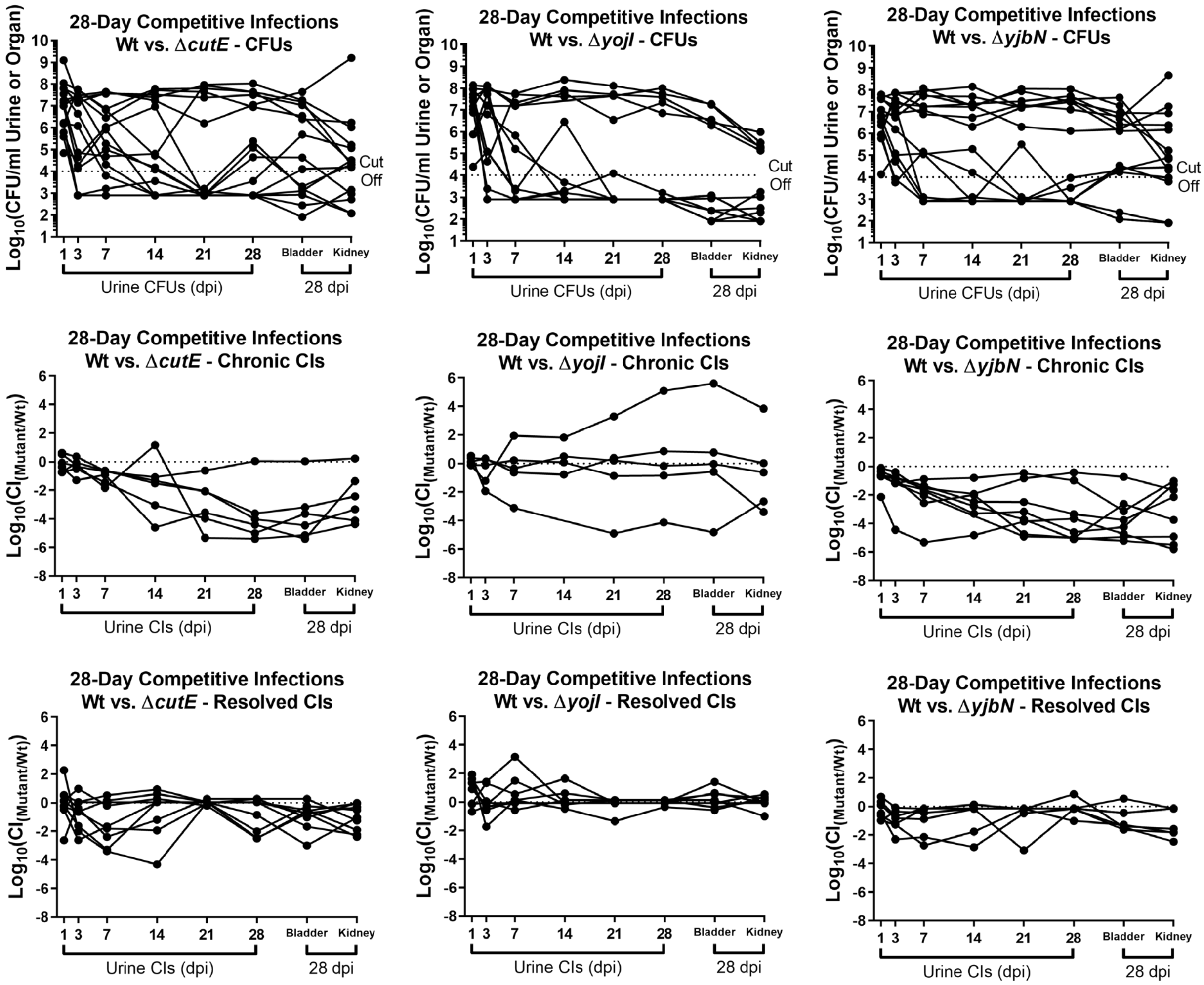
28-Day Competitive Infections
Wt vs. ΔcutE - CFUs
28-Day Competitive Infections
Wt vs. ΔyojI - CFUs
28-Day Competitive Infections
Wt vs. ΔyjbN - CFUs
Log10(CFU/ml Urine or Organ)
Cut Off
Urine CFUs (dpi)
28 dpi
Bladder
Kidney
28-Day Competitive Infections
Wt vs. ΔcutE - Chronic CIs
28-Day Competitive Infections
Wt vs. ΔyojI - Chronic CIs
28-Day Competitive Infections
Wt vs. ΔyjbN - Chronic CIs
Log10(CI(Mutant/Wt))
Urine CIs (dpi)
28-Day Competitive Infections
Wt vs. ΔcutE - Resolved CIs
28-Day Competitive Infections
Wt vs. ΔyojI - Resolved CIs
28-Day Competitive Infections
Wt vs. ΔyjbN - Resolved CIs

Figure S1x.

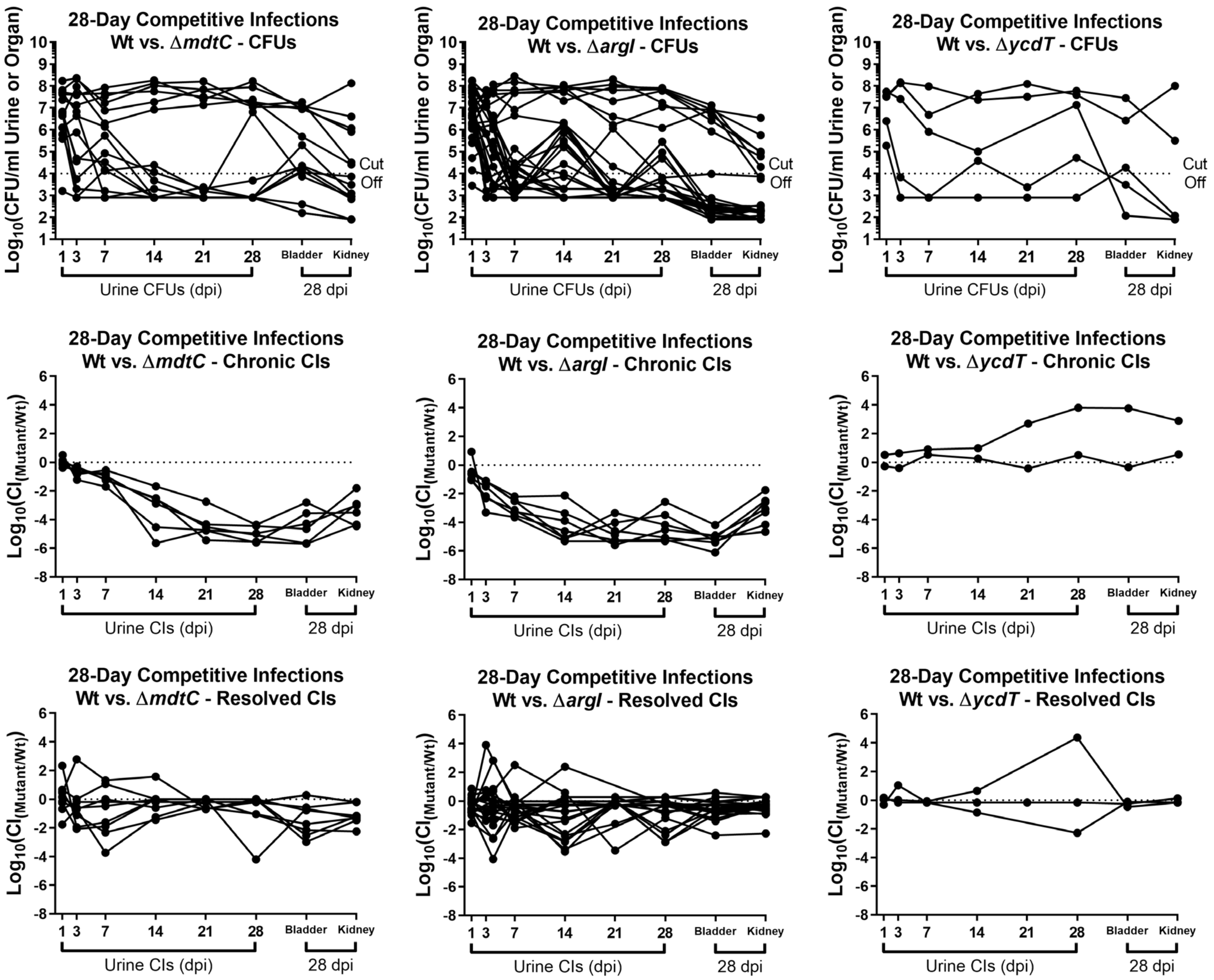
28-Day Competitive Infections
Wt vs. ΔmdtC - CFUs
Log10(CFU/ml Urine or Organ)
Cut Off
Urine CFUs (dpi)
28 dpi
Bladder
Kidney
28-Day Competitive Infections
Wt vs. ΔargI - CFUs
28-Day Competitive Infections
Wt vs. ΔycdT - CFUs
28-Day Competitive Infections
Wt vs. ΔmdtC - Chronic CIs
Log10(CI(Mutant/Wt))
Urine CIs (dpi)
28-Day Competitive Infections
Wt vs. ΔargI - Chronic CIs
28-Day Competitive Infections
Wt vs. ΔycdT - Chronic CIs
28-Day Competitive Infections
Wt vs. ΔmdtC - Resolved CIs
28-Day Competitive Infections
Wt vs. ΔargI - Resolved CIs
28-Day Competitive Infections
Wt vs. ΔycdT - Resolved CIs
