# Supplementary figures and images for: Adaptation of Arginine Synthesis among Uropathogenic Branches of the Escherichia coli Phylogeny Reveals Adjustment to the Urinary Tract Habitat
Source: mBio. 2020 Sep 29;11(5):e02318-20. doi: 10.1128/mBio.02318-20 (PMC7527732; doi:10.1128/mBio.02318-20)

Figure S2.

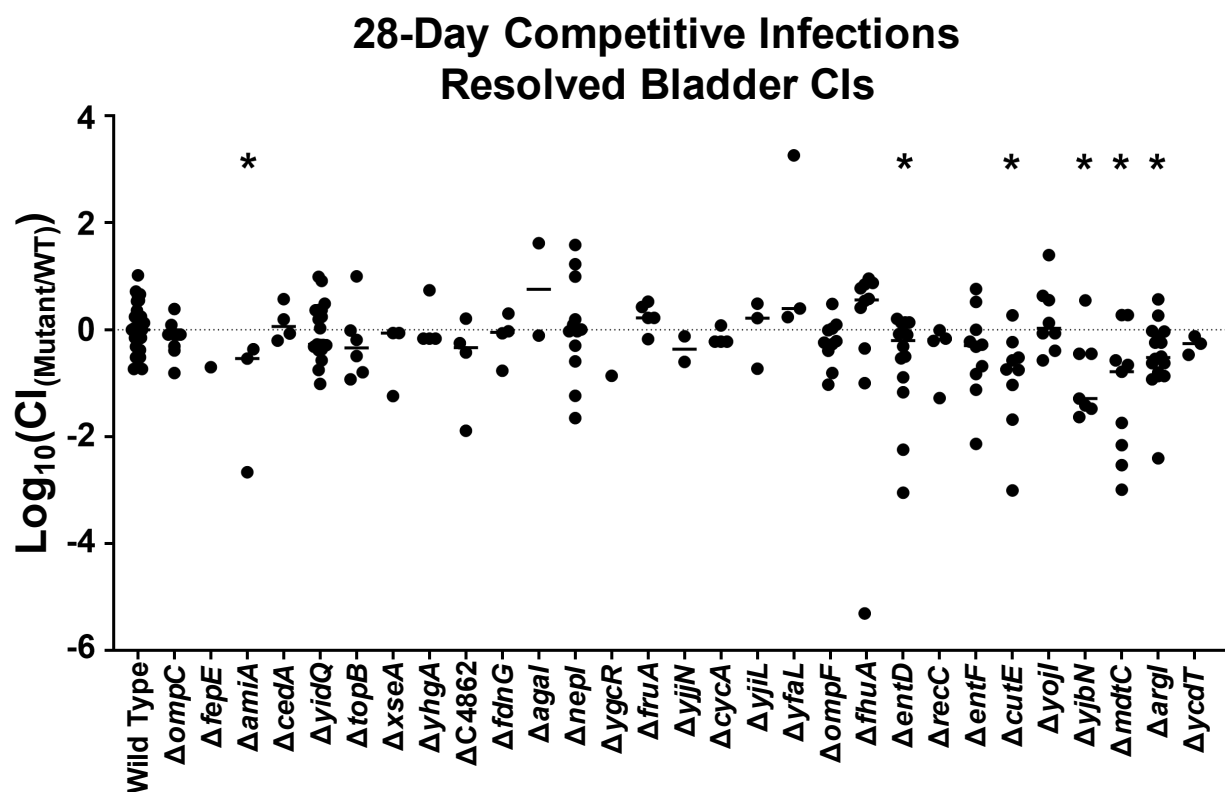

Supplement: FIG S2 [file mBio.02318-20-sf002.pdf]

Figure S3.

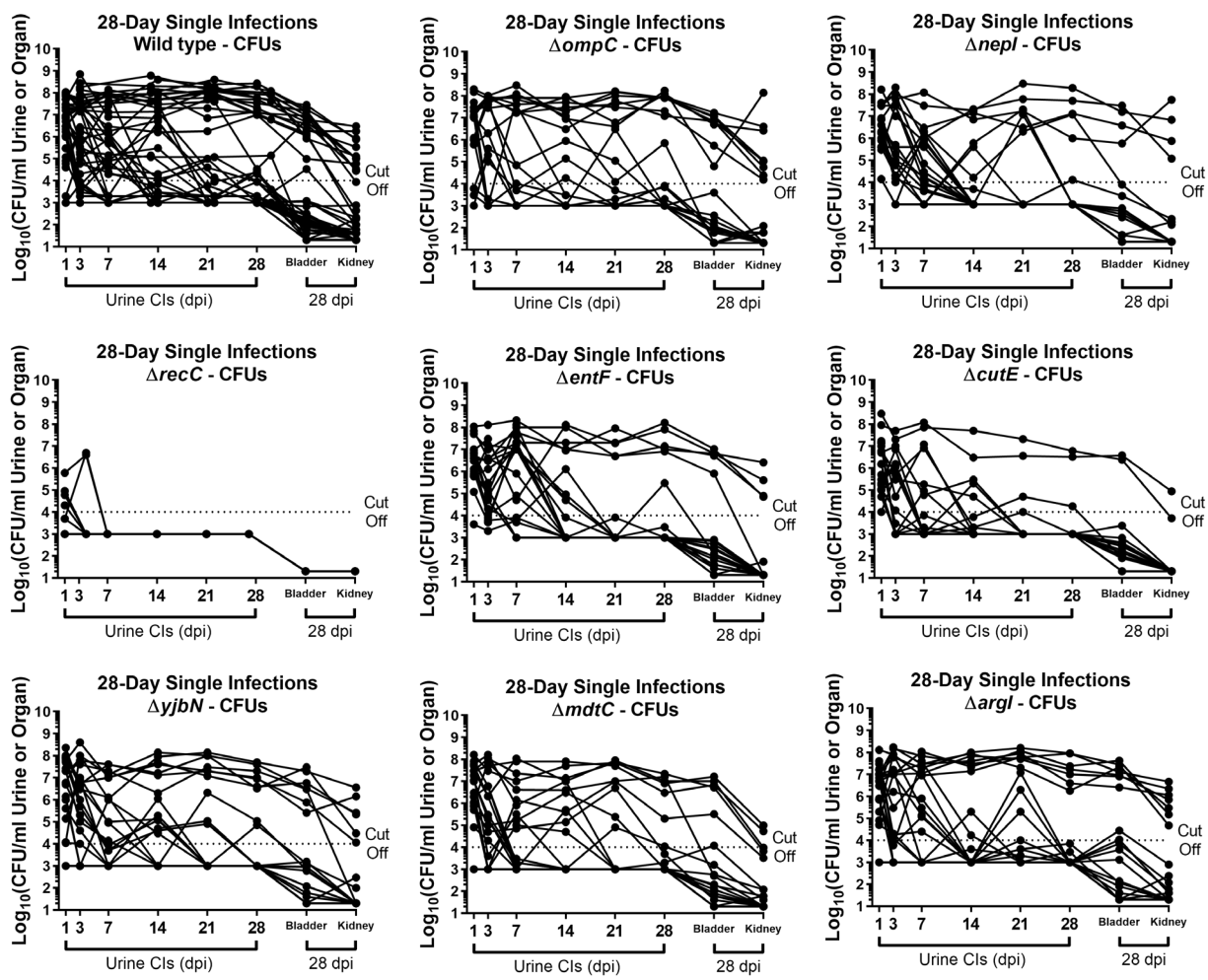

Supplement: FIG S3 [file mBio.02318-20-sf003.pdf]

Figure S4.

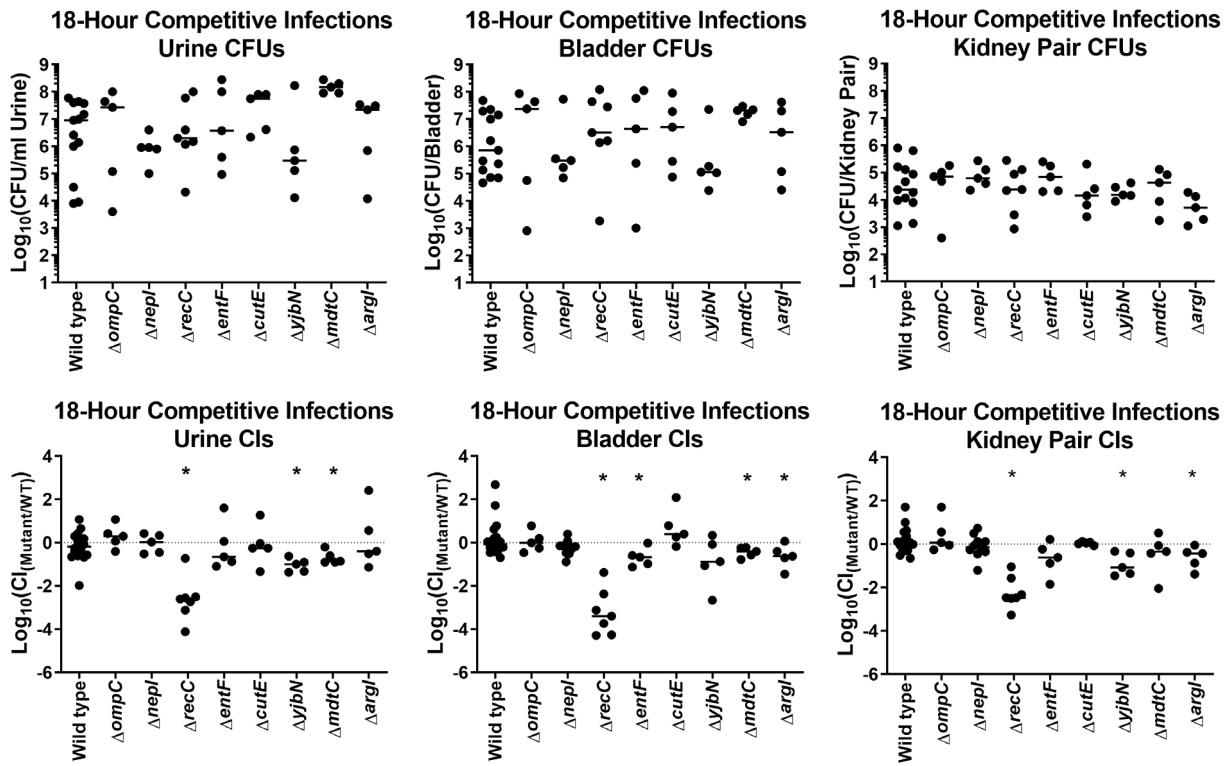

Supplement: FIG S4 [file mBio.02318-20-sf004.pdf]

Figure S5.

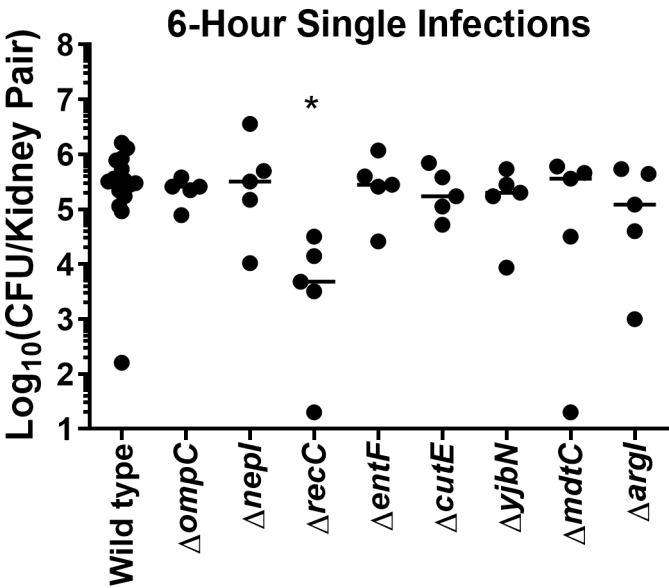

Supplement: FIG S5 [file mBio.02318-20-sf005.pdf]

Figure S6.

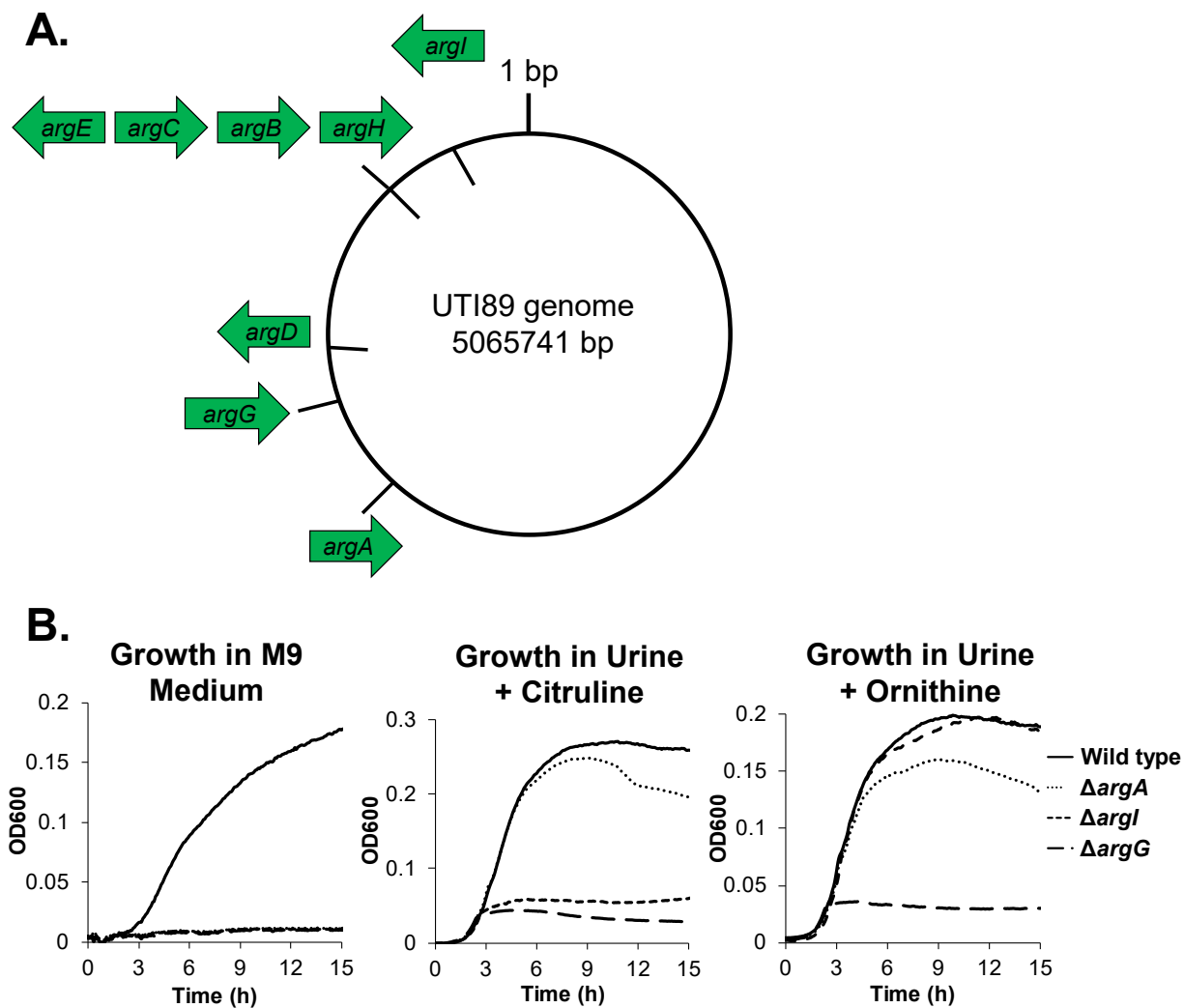

Supplement: FIG S6 [file mBio.02318-20-sf006.pdf]

Figure S7.

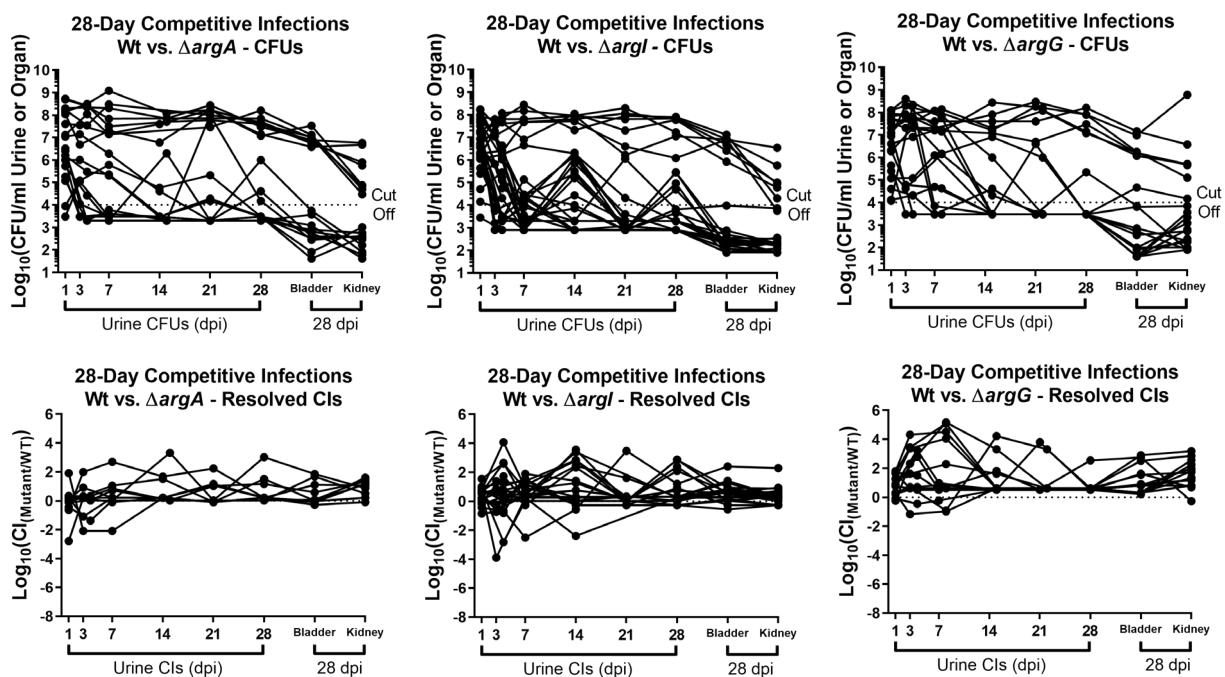

Supplement: FIG S7 [file mBio.02318-20-sf007.pdf]

Figure S8.

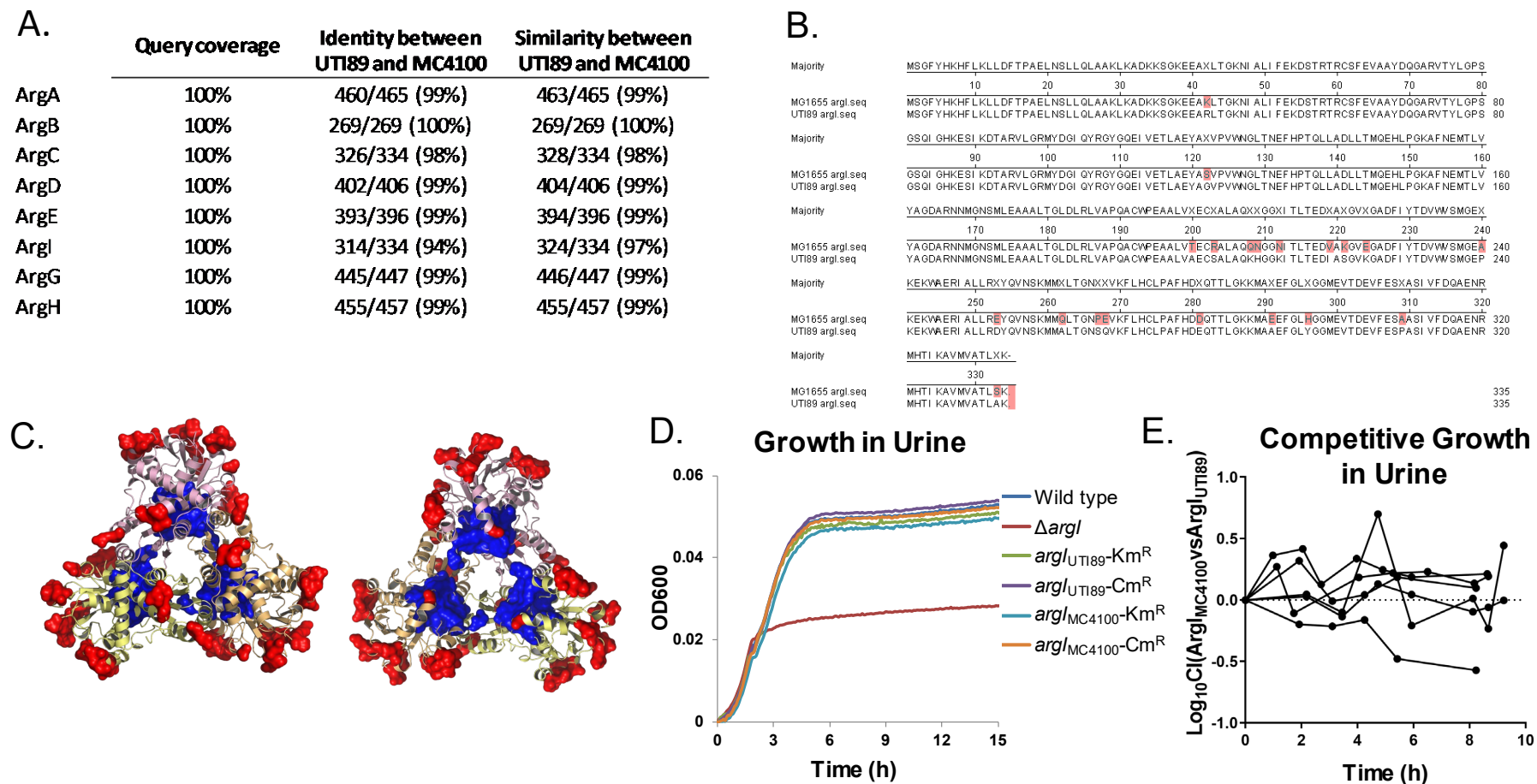

Supplement: FIG S8 [file mBio.02318-20-sf008.pdf]

Figure S9.

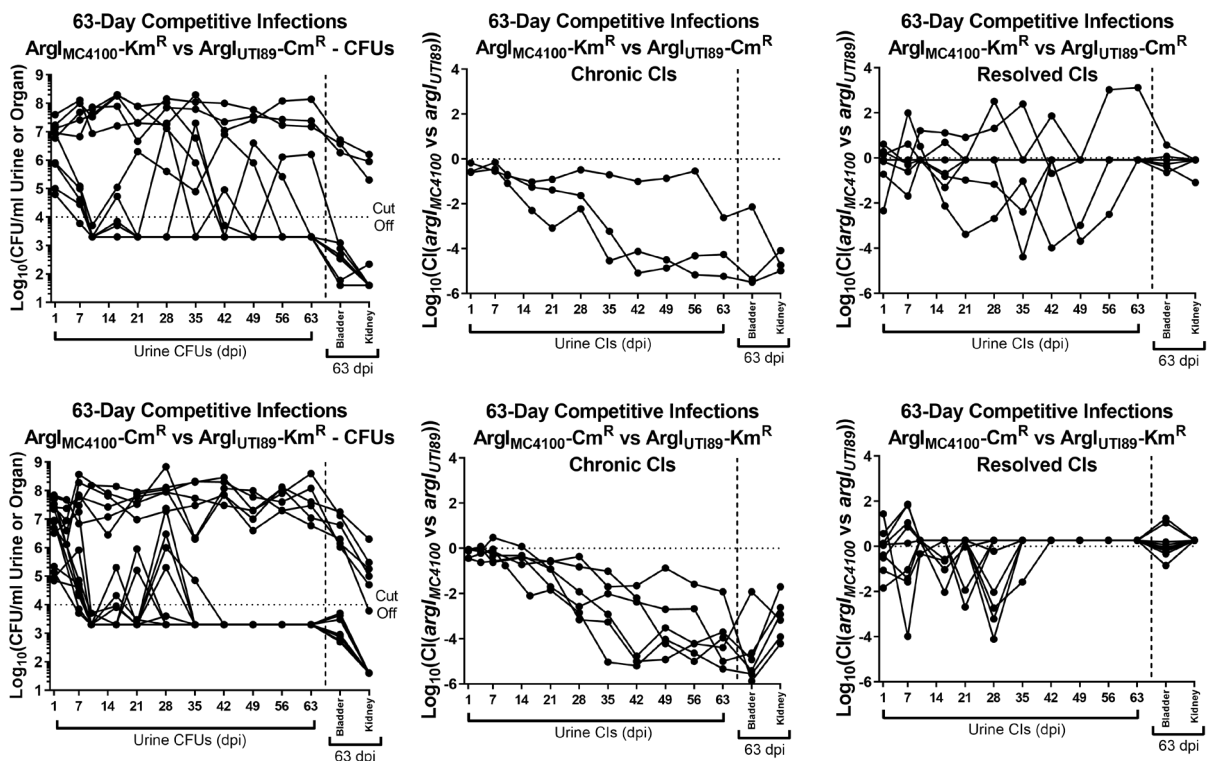

Supplement: FIG S9 [file mBio.02318-20-sf009.pdf]
