# Supplementary material for: Adaptation of Arginine Synthesis among Uropathogenic Branches of the Escherichia coli Phylogeny Reveals Adjustment to the Urinary Tract Habitat
Source: mBio. 2020 Sep 29;11(5):e02318-20. doi: 10.1128/mBio.02318-20 (PMC7527732; doi:10.1128/mBio.02318-20)
Supplement: TABLE S1 [file mBio.02318-20-st001.docx]

**Table S1. Total numbers of mice and frequency of outcome of 28-day competitive infections.**

| **Wild type**  **vs.** | **Total**  **Mice** | **Percent**  **Chronic** | **Percent**  **Resolved** |
| --- | --- | --- | --- |
| Wild type | 53 | 45 | 55 |
| Δ*ompC* | 15 | 40 | 60 |
| Δ*fepE* | 5 | 80 | 20 |
| Δ*amiA* | 5 | 40 | 60 |
| Δ*cedA* | 5 | 20 | 80 |
| Δ*yidQ* | 20 | 5 | 95 |
| Δ*topB* | 15 | 60 | 40 |
| Δ*xseA* | 5 | 40 | 60 |
| Δ*yhgA* | 5 | 20 | 80 |
| Δ*C4862* | 5 | 20 | 80 |
| Δ*fdnG* | 5 | 20 | 80 |
| Δ*agal* | 5 | 60 | 40 |
| Δ*nepI* | 23 | 48 | 52 |
| Δ*ygcR* | 5 | 80 | 20 |
| Δ*fruA* | 6 | 17 | 83 |
| Δ*yjjN* | 5 | 60 | 40 |
| Δ*cycA* | 5 | 20 | 80 |
| Δ*yjiL* | 5 | 40 | 60 |
| Δ*yfaL* | 4 | 25 | 75 |
| Δ*ompF* | 15 | 33 | 67 |
| Δ*fhuA* | 15 | 33 | 67 |
| Δ*entD* | 20 | 30 | 70 |
| Δ*recC* | 10 | 60 | 40 |
| Δ*entF* | 19 | 47 | 53 |
| Δ*cutE* | 15 | 40 | 60 |
| Δ*yojI* | 13 | 38 | 62 |
| Δ*yjbN* | 15 | 53 | 47 |
| Δ*mdtC* | 15 | 40 | 60 |
| Δ*argl* | 23 | 30 | 70 |
| Δ*ycdT* | 5 | 40 | 60 |
